# Analysis of the DNA‐binding properties of TGF‐β‐activated Smad complexes unveils a possible molecular basis for cellular context‐dependent signaling

**DOI:** 10.1096/fj.202400978R

**Published:** 2024-08-08

**Authors:** Yuka Itoh, Kunio Miyake, Daizo Koinuma, Chiho Omata, Masao Saitoh, Keiji Miyazawa

**Affiliations:** ^1^ Department of Biochemistry, Graduate School of Medicine University of Yamanashi Yamanashi Japan; ^2^ Department of Epidemiology and Environmental Medicine, Graduate School of Medicine University of Yamanashi Yamanashi Japan; ^3^ Department of Pathology, Graduate School of Medicine The University of Tokyo Tokyo Japan; ^4^ Center for Medical Education and Sciences, Graduate School of Medicine University of Yamanashi Yamanashi Japan

**Keywords:** context‐dependent signaling, Smad, TGF‐β

## Abstract

Transforming growth factor‐β (TGF‐β) is a pleiotropic cytokine that modulates a wide variety of cellular responses by regulating target gene expression. It principally transmits signals via receptor‐activated transcription factors Smad2 and Smad3, which form trimeric complexes with Smad4 upon activation and regulate gene expression by binding to genomic DNA. Here, we examined the mechanisms by which TGF‐β regulates the transcription of target genes in a cell context‐dependent manner by screening a double‐stranded DNA oligonucleotide library for DNA sequences bound to endogenous activated Smad complexes. Screening was performed by cyclic amplification of selected targets (CASTing) using an anti‐Smad2/3 antibody and nuclear extracts isolated from three cell lines (A549, HepG2, and HaCaT) stimulated with TGF‐β. The preference of the activated Smad complexes for conventional Smad‐binding motifs such as Smad‐binding element (SBE) and CAGA motifs was different in HepG2 than in the other two cell lines, which may indicate the distinct composition of the activated Smad complexes. Several transcription factor‐binding motifs other than SBE or CAGA, including the Fos/Jun‐binding motifs, were detected in the enriched sequences. Reporter assays using sequences containing these transcription factor‐binding motifs together with Smad‐binding motifs indicated that some of the motifs may be involved in cell type‐dependent transcriptional activation by TGF‐β. The results suggest that the CASTing method is useful for elucidating the molecular basis of context‐dependent Smad signaling.

## Graphical abstract

To elucidate a molecular basis for context‐dependent TGF‐β signaling, DNA sequences bound to endogenous Smad2/3 were concentrated from a double‐stranded DNA oligonucleotide library by cyclic amplification of selected targets (CASTing). In addition to Smad‐binding sequences, binding motifs to candidate Smad cofactors (transcription factors that cooperate with Smad proteins) were detected in the enriched sequences. Some of the enriched sequences responded to TGF‐β stimulation in reporter assays, suggesting that the CASTing method is useful for identifying novel Smad cofactors.

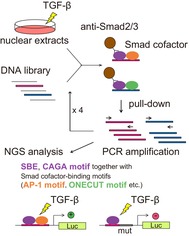

**Abbreviations**
AP‐1: activator protein‐1
CASTing: cyclic amplification of selected targets
SBE: Smad‐binding element
TGF‐β: transforming growth factor‐β

## 1 INTRODUCTION

Transforming growth factor‐β (TGF‐β) is a pleiotropic cytokine that plays crucial roles in embryonic development and adult tissue homeostasis.^1^ TGF‐β and its receptors are widely expressed throughout the body. The specificity of its action is, in part, mediated by context‐dependent intracellular signaling pathways, which are classified into Smad‐dependent and non‐Smad pathways.^2^, ^3^ Smad proteins are activated by ligand‐stimulated receptors and transmit signals principally by regulating the transcription of target genes through direct association with genomic DNA.^4^, ^5^, ^6^, ^7^

Among eight Smad family proteins identified in mammals, Smad2 and Smad3 phosphorylation is induced by TGF‐β via a heteromeric complex composed of type I and type II receptors. Phosphorylated Smad2 or Smad3 then forms a trimeric complex with Smad4, which translocates to the nucleus and positively or negatively regulates the transcription of target genes. Although Smad3 and Smad4 possess intrinsic ability to interact with DNA, their affinities are weak (K_d_, 100–300 nM).^8^ This is because the core elements of the Smad‐binding motifs consist of only 4 bp, that is, 5′‐GTCT‐3′ or its complementary 5′‐AGAC‐3′. Thus, the activated Smad complex requires the assistance of other transcription factors, collectively termed “Smad cofactors,” to ensure the stability and selectivity of DNA binding.^6^ The profiles of Smad cofactors that function in target cells are thought to shape context‐dependent target gene expression. In addition, variation in the composition of Smad trimeric complexes, which is determined by the expression levels of each Smad protein, affects cell responses to TGF‐β.^9^, ^10^, ^11^, ^12^, ^13^ This could be another mechanism underlying context‐dependent Smad signaling.

In this study, we examined the DNA‐binding properties of complexes formed by endogenous Smad proteins isolated from the nuclear extracts of three cell lines (A549, HepG2, and HaCaT) stimulated with TGF‐β. Binding sequences recognized by Smad complexes exhibited unique signatures in different cell lines, which could contribute to context‐dependent Smad signaling. We found that enriched sequences contain the binding motifs of several candidate Smad cofactors, in addition to conventional Smad‐binding motifs, Smad‐binding element (SBE, 5′‐GTCTAGAC‐3′) or CAGA motifs (5′‐CCAGACA‐3′ or 5′‐TGTCTGG‐3′). The ability of these sequences to respond to TGF‐β was examined using luciferase reporter assays. Although some of the sequences, including those containing Fos/Jun‐binding motifs, responded to TGF‐β, their mutant versions did not, suggesting that they can be used to monitor Smad cofactor‐dependent gene expression. Biochemical characterization of the transcriptional responses of DNA sequences with composite SBE and the Fos/Jun‐binding motifs showed that these sequences respond to TGF‐β stimulation differently in various target cells. The results indicate that the current strategy is useful to explore context‐dependent Smad signaling.

## 2 MATERIALS AND METHODS

### 2.1 Cell lines and genome editing using the CRISPR/Cas9 system

HaCaT cells (RRID:CVCL_0038) were a gift from Dr. N. E. Fusenig (German Cancer Research Center, Heidelberg, Germany). HepG2/C3A (RRID:CVCL_1098), A549 (RRID:CVCL_0023), and NMuMG normal murine mammary gland epithelial cells (RRID:CVCL_0075) were obtained from the American Type Culture Collection. Human cells (HaCaT, HepG2/C3, and A549) were authenticated by short tandem repeat analysis. *SMAD3*‐knockout (A549‐S3KO), *SMAD2*‐knockout (A549‐S2KO), *SMAD2/3*‐double knockout (A549‐S2/3DKO), and *SMAD4*‐knockout (A549‐S4KO) A549 cells were described previously.^14^ *SMAD2/3/4*‐triple knockout cells (A549‐S2/3/4 TKO) were established from A549‐S2KO cells using CRISPR plasmids, sc‐400069‐NIC‐2 (Santa Cruz Biotechnology) and pDG462‐SMAD4 (expression for hSpCas9 and 2 gRNAs, 5′‐CTATGCACAATGCTCAGAC‐3′ and 5′‐ TTGATGTGCCATAGACAAGG‐3′). pDG462 was a gift from Prof. Paul Thomas (Addgene plasmid #100903; http://n2t.net/addgene:100903; RRID:Addgene_100903).^15^ The deletion/disruption of target genes was confirmed by immunoblotting and genomic sequencing (Figure S1). All cells were maintained in Dulbecco's modified Eagle's medium containing 10% fetal bovine serum, 50 units/mL penicillin, and 50 μg/mL streptomycin. For culturing NMuMG cells, the culture medium was supplemented with insulin (10 μg/mL).

### 2.2 Antibodies and ligands

Anti‐Smad4 (D3R4N) antibody was purchased from Cell Signaling Technology. Anti‐Smad2/3 antibody (610843) was purchased from BD Biosciences. Anti‐JunB antibody (C‐11) was obtained from Santa Cruz Biotechnology. Anti‐phospho‐Smad2 (Ser465/467) (A5S) and anti‐α‐tubulin (DM1A) antibodies were purchased from Sigma‐Aldrich. Human recombinant TGF‐β1 (100‐21) was purchased from PeproTech.

### 2.3 Cyclic amplification of selected targets (CASTing)

The collection of endogenous‐activated Smad complex‐binding DNA sequences was performed essentially as described previously.^16^ An oligonucleotide library containing 30 random nucleotides flanked by sequences for PCR amplification (5′‐GCGTCGACTAGATCTGCAG‐(N_30_)‐GAATTCGGATCCCTCGAGCG‐3′) was synthesized and used for screening. HaCaT, HepG2/C3A, and A549 cells were stimulated with TGF‐β1 (1 ng/mL) for 1 h and harvested. Nuclear extracts were prepared using the NucBuster Protein Extraction Kit (Millipore), followed by CASTing analysis^17^ using an anti‐Smad2/3 antibody. Taq polymerase (Takara Bio) was used for DNA amplification. In each round of selection, 50–70 μg of protein was used. After four rounds of selection, amplified DNA fragments were subjected to sequencing using the Ion Torrent and Ion Plus Fragment Library Kit (Life Technologies). Raw data were deposited to the DDBJ BioProject database (PRJDB9205).

### 2.4 CASTing data analysis

Collected sequences were clustered using Aptamer Clustering_2.6.1 (Life Technologies) and sorted into sequences containing SBE or CAGA‐motifs. A total of 1000 clustered sequences were analyzed using MEME version 5.2.0 (RRID:SCR_001783) with the following parameters: number of motifs = 100, width of motifs = 5–12, site of motif = 3–600, and other locations at default.^18^ The best possible match sequences in the motifs were identified using MAST (RRID:SCR_016340),^19^ and transcription factors with the most significant match sequences (*E* < .05) were obtained using Tomtom.^20^

### 2.5 ChIP‐chip or ChIP‐seq data analysis

Smad2/3‐binding sequences from ChIP‐chip in HaCaT cells (GSE11710)^21^ and HepG2 cells (GSE28798),^22^ and Smad3‐binding sequences from ChIP‐seq in A549 cells (GSE51510)^23^ were used. Peaks were called using CisGenome version 2 (RRID:SCR_001558) (two‐sample analysis with an FDR cutoff of 0.05),^24^ and 1000‐bp long genomic sequences flanking the middle position were obtained using CisGenome. A set of sequences with individual motif occurrence was scanned using FIMO.^25^

### 2.6 Luciferase assay

Luciferase assay was performed as described previously.^16^ In brief, cells were transfected with the indicated luciferase reporter constructs and the pRL‐TK vector using PEI Max transfection reagent (Polysciences), stimulated with TGF‐β1 (1 ng/mL) for 18 h, and harvested. Luciferase activity was measured using the dual‐luciferase reporter system (Promega) and a luminometer (Spectra Max L, Molecular Devices). Values were normalized to those measured for Renilla luciferase under the control of the thymidine kinase promoter. Luciferase activity is expressed as fold induction by TGF‐β unless otherwise noted. All the reporter constructs used in this study have the pGL4‐MLP backbone (pGL4 vector containing the TATA box and the initiator sequence of the adenovirus major late promoter). CAGA_12_‐MLP‐Luc and SSS‐Luc were described previously.^16^ Oligonucleotides for artificial reporter constructs were synthesized with flanking *Kpn*I and *Xho*I sites, and inserted into pGL4‐MLP. Sequences for artificial reporter constructs are listed in Table 1.

**TABLE 1:** Nucleotide sequences of artificial reporter constructs.

Name	Sequence
SSS‐Luc	AT **GTCTAGAC** AAT **GTCTAGAC** AAT **GTCTAGAC** A
AP1‐1	TTCATACT **GTCTAGAC** TGCTGA **TGACTCA** TCGTCTG
AP1‐2	TTCCTAGCGA **TGAGTCA** CGCGTCT **GTCTAGAC** ACTG
AP1‐3	TTCTGT **GTCTAGAC** ATGCGA **TGAGTCA** TACGCCCTG
AP1‐4	TTCTACAA **TGACTCA** TCGCT **GTCTAGAC** AACGGCTG
AP1‐2 mSBE	TTCCTAGCGA **TGAGTCA** CGCGTCT ** ATGTACAT ** ACTG
AP1‐2 mAP‐1	TTCCTAGCGA ** CA ** **AGTCA** CGCGTCT **GTCTAGAC** ACTG
AP1‐2 mSBE/mAP‐1	TTCCTAGCGA ** CA ** **AGTCA** CGCGTCT ** ATGTACAT ** ACTG
AP1‐2m4	CTAGCGA **TGAGTCA** CGCGTCT **GTCTAGAC** A
AP1‐2m4‐D2, AP1‐SBE‐Luc	CGA **TGAGTCA** CGCGTCT **GTCTAGAC** A
AP1‐2m4‐D3	**TGAGTCA** CGCGTCT **GTCTAGAC** A
AP1‐SBE‐AC	CGA **TGAGTCA** CGCGTCTAG **CCAGACA**
AP1‐SBE‐ACc	CGA **TGAGTCA** CGCGTC **TGTCTGG** CTA
AP1‐SBE‐Cc3	CGA **TGAGTCA** CGCGTC **TGTCTGG** CTAAA **TGTCTGG** CTTTT **TGTCTGG** CT
AP1‐SBE‐AA	CGA **TGAGTCA** CGCGTCTA **TGAGTCA** C
AP1‐SBE‐SA	CGT **GTCTAGAC** AGCGTCA **TGAGTCA** C
AP1‐SBE‐SS	CGT **GTCTAGAC** ACGTCT **GTCTAGAC** A
AP1‐SBE‐dLinker‐1	CGA **TGAGTCA** CGCT **GTCTAGAC** A
AP1‐SBE‐dLinker‐2	CGA **TGAGTCA** GTCT **GTCTAGAC** A
AP1D1‐S‐Luc	TTCCT **GTCTAGAC** AGCGA **TGAATCATC** GATCGCCTG
AP1D1‐S‐Luc mAP‐1	TTCCT **GTCTAGAC** AGCGA ** CA ** **AATCATC** GATCGCCTG
AP1D2‐CS‐Luc	TTC **GTCTAGAC** ATA **TGTCTGG** C **TGATTCATC** GCCTG
AP1D2‐CS‐Luc mAP‐1	TTC **GTCTAGAC** ATA **TGTCTGG** C ** CA ** **ATTCATC** GCCTG
1. HepG2‐S56(ONECUT1)	TTC **AAATCGAT** AT **GTCTAGAC** ACACCGTCTGGGCTG
mut. HepG2‐S56(ONECUT1)	TTC **AAACCGGT** AT **GTCTAGAC** ACACCGTCTGGGCTG
2. HaCaT‐21(SOX13)	T **TCCACTGTT** AAT **GTCTAGAC** AGAC **TGTCTGG** CCTG
mut. HaCaT‐21(SOX13)	T **TCCAAAAA ** TAAT **GTCTAGAC** AGAC **TGTCTGG** CCTG
3. A549‐C51(ZNF435)	TTCC **GGTGTTCTG** CT **GTCTAGAC** AC **TGTCTGG** CCTG
mut. A549‐C51(ZNF435)	TTCC ** A A A A A A A A A ** CT **GTCTAGAC** AC **TGTCTGG** CCTG
4. A549‐C99(PRDM4)	TTCCT **GTCTAGAC** AC **TGTCTGG** ATCCTT **GGGGCCT** G
mut. A549‐C99(PRDM4)	TTCCT **GTCTAGAC** AC **TGTCTGG** ATCCTT **GCGCCTT** G
5. A549‐C94(ASCL1)	CAGACGC **AGCTGCT** G **CCAGACA** GT **GTCTAGAC** CGA
mut. A549‐C94(ASCL1)	CAGACG ** TGGCCTCT** G **CCAGACA** GT **GTCTAGAC** CGA
6. HepG2‐C58(ZNF306)	TTC **GTCTAGAC** ACT **TGTCTGG** CGTTC **GGCTAGCCT** G
mut. HepG2‐C58(ZNF306)	TTC **GTCTAGAC** ACT **TGTCTGG** CGTTC ** TGCTTGTGA ** G
7. HaCaT‐S46(SOX14)	TTC **TAACATTGA** T **GTCTAGAC** AC **TGTCTGG** CCGCTG
mut. HaCaT‐S46(SOX14)	TTC**TCGAAGAGA**T **GTCTAGAC** AC **TGTCTGG** CCGCTG
8. HaCaT‐C88(MTF1)	TTCTGG **TGTCTGG** T **TTTGCACAC GTCTAGAC** AGCTG
mut. HaCaT‐C88(MTF1)	TTCTGG **TGTCTGG** T **TTAAAAAAC GTCTAGAC** AGCTG
9. HaCaT‐S18(NFIX)	TTCGAAAG **CCGTGCCAG** CCT **GTCTAGAC** ACTGTCTG
mut. HaCaT‐S18(NFIX)	TTCGAAAG **CCGTATTAG** CCT **GTCTAGAC** ACTGTCTG
10. HaCaT‐73(Bhlha15)	TTCGTCT **GTCTAGAC** ACTGTCTAGT **TCATATGG** CTG
mut. HaCaT‐73(Bhlha15)	TTCGTCT **GTCTAGAC** ACTGTCTAGT **TAAAAAAA ** CTG
11. HepG2‐25(E2F1)	TTCGTAGACGTTC **GTCTAGAC** AC **ATTGGCGCC** CCTG
mut. HepG2‐25(E2F1)	TTCGTAGACGTTC **GTCTAGAC** AC **ATTGGATAC** CCTG
12. A549‐92(Gm397)	TTCGAGT **GTCTAGAC** AA **TGTCTGG** GC **GTGTGTGC** TG
13. A549‐C62(MYC)	TTCGC **GTCTAGAC** GG **TGTCTGG** ACG **GCACGTGGCT** G
14. A549‐27(SOX10)	T **TCGCTGCTATTG TCTAGAC** A **TGTCTGG** TCTGGCTG
15. HepG2‐58(Tcfe2a)	TTCTCTAGACATA **GTCTAGAC** TGGC **ATCTGGCCT** G
16. HepG2‐65(ZNF306)	T **TCGAGGCTA** AAA **GTCTAGAC** GGTCGTCTGGAGCTG
17. HepG2‐37(REST)	TTCGCTAGACACT **GTCTAGAC** ATGTGG **ATGGTGCT** G

*Note*: These sequences were synthesized with flanking *Kpn*I and *Xho*I sites and inserted into the pGL4‐MLP vector. SBE (GTCTAGAC), CAGA motif (TGTCTGG), and the AP‐1‐binding motif (TGA(G/C)TCA or TGADTCATC, D = G/A/T) are colored *red*, *blue*, and *purple*, respectively. Putative transcription factor‐binding motifs and their mutant sequences are colored *green* and *gray*, respectively. Mutated residues are underlined.

### 2.7 DNA affinity precipitation assay

DNA affinity precipitation assay was performed as described previously^16^ with modifications. NMuMG cells were stimulated with TGF‐β1 (1 ng/mL) for 4 h and harvested. Cell lysates (200–300 μg protein) were added to DNA‐bound Dynabeads M280 streptavidin (Life Technologies) in the presence of salmon sperm DNA (80 μg/mL) (FUJIFILM Wako Pure Chemical Corporation) and incubated overnight at 4°C. Dynabeads were washed, and bound proteins were separated by SDS‐PAGE and detected by immunoblotting. A sequence of a biotinylated probe is 5′‐TCGAGCGATGAGTCACGCGTCTGTCTAGACAATG‐3′.

### 2.8 Statistical analysis

The Tukey's multiple comparison test was used to determine significant differences between WT reporter groups and mutant reporter groups in TGF‐β‐stimulated cells. Statistical calculations were performed using R (version 4.2.1). *p* < .05 indicated statistical significance.

## 3 RESULTS

### 3.1 DNA binding properties of the TGF‐β–activated Smad complexes isolated from A549, HepG2, and HaCaT cells

We previously performed CASTing analysis to determine the DNA‐binding properties of Smad3 and Smad4, which are overexpressed in 293T cells.^16^ We found that although both Smad3 and Smad4 interact with SBE and CAGA motifs, Smad3 shows a preference for CAGA motifs over SBE, whereas Smad4 shows the opposite pattern. In this study, we used a similar strategy to determine the properties of activated Smad complexes in different cell lines by identifying the DNA sequences bound to endogenous Smad complexes activated by TGF‐β in three target cell lines: A549 (human lung adenocarcinoma), HepG2 (human hepatoblastoma), and HaCaT (human normal immortalized keratinocytes).

Cells were stimulated with TGF‐β for 1 h, and nuclear extracts were prepared and subjected to CASTing analysis using an anti‐Smad2/3 antibody and a double‐stranded oligonucleotide library containing 30 random nucleotides. The length of random sequences in the library was selected to contain two or three transcription factor‐binding motifs. The Smad complex‐binding sequences were enriched by four rounds of selection and analyzed using a next‐generation sequencer. The occurrence of Smad‐binding motifs in A549, HepG2, and HaCaT cells is shown in Table 2. In the three cell lines, an 8‐bp palindromic SBE (5′‐GTCTAGAC‐3′) was the most frequent site, whereas the CAGA motifs (5′‐CCAGACA‐3′ or 5′‐TGTCTGG‐3′) were less frequent among DNA‐binding sequences interacting with activated Smad complexes isolated from HepG2 cells. Five‐base pair GC motifs (5′‐GGCGC‐3′, 5′‐GCGCC‐3′, 5′‐GGCCG‐3′, and 5′‐CGGCC‐3′) were previously identified as Smad‐binding motifs.^26^ The 5‐bp GC motifs were also found in the Smad complex‐binding sequences identified in the three cell lines. However, they occurred at a lower frequency than the SBE.

**TABLE 2:** Occurrence of Smad‐binding motifs among the binding sequences for activated Smad complexes in TGF‐β–stimulated A549, HepG2, and HaCaT cells.

	Total reads	SBE	CAGA	5‐bp GC
A549	205 896	113 290 (550)	33 360 (162)	29 325 (142)
HepG2	186 760	98 925 (530)	14 443 (77)	34 241 (183)
HaCaT	261 925	182 396 (696)	60 508 (231)	37 344 (143)
293T/Smad3	120 906	56 859 (470)	87 563 (724)	8469 (70)
293T/Smad4	106 048	88 098 (831)	17 414 (164)	5877 (55)

*Note*: Binding sequences to endogenous activated Smad complexes in A549, HepG2, and HaCaT cells stimulated by TGF‐β were concentrated from a double‐stranded DNA oligonucleotide library with 30‐nucleotide random sequences using an anti‐Smad2/3 antibody. The occurrence of SBE (5′‐GTCTAGAC‐3′), CAGA motifs (5′‐CCAGACA‐3′ and 5′‐TGTCTGG‐3′), and 5‐bp GC motifs (5′‐GGCGC‐3′, 5′‐GCGCC‐3′, 5′‐GGCCG‐3′, and 5′‐CGGCC‐3′) among total reads are shown. The occurrence of each motif per 1000 reads is shown in parentheses. Data on 293T/Smad3 and 293T/Smad4 (293T cells exogenously overexpressing either FLAG‐tagged Smad3 or Smad4, immunoprecipitated by anti‐FLAG M2 magnetic beads) are cited from Ref. [16].

We next clustered the collected sequences from three cell lines. The top 30 concentrated sequences are shown in Table S1. Most of these sequences contain two SBEs or one SBE plus one CAGA motif. Many top 30 binding sequences in HepG2 cells contained two SBEs but no CAGA motif, similar to the pattern observed in the top 30 binding sequences interacting with Smad4 overexpressed in HEK293T cells.^16^ This trend is consistent with the occurrence of SBE and CAGA motifs in Smad complex‐binding sequences obtained from HepG2 cells (Table 2). These results suggest that the composition of activated Smad complexes varies among different target cells, which may reflect the different expression levels of each Smad protein^13^ or the presence of additional modulatory factor(s) in the complexes.

### 3.2 Reporter constructs containing exclusively SBE or CAGA motifs exhibit different extents of activation according to cell type

**FIGURE 1:**
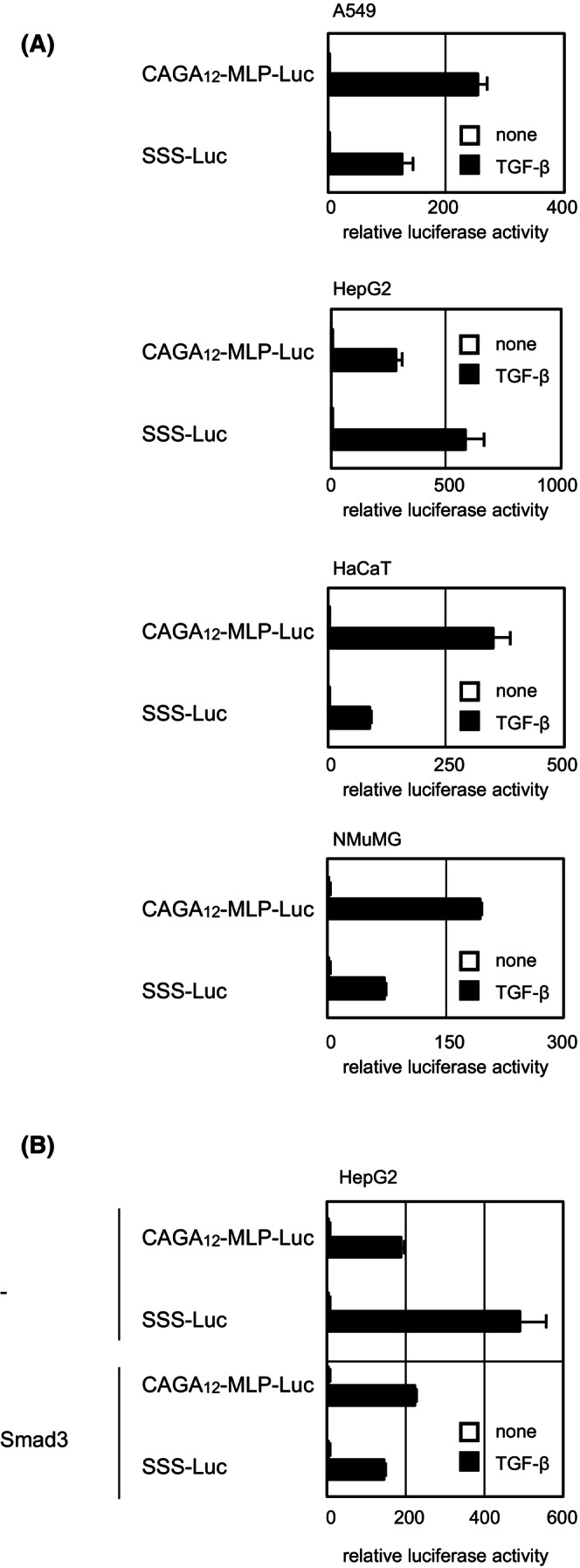
TGF‐β‐induced activation of CAGA or SBE‐based artificial reporters in various target cells. (A) CAGA_12_‐MLP‐Luc or SSS‐Luc was transiently transfected with the control *Renilla* luciferase plasmid into the indicated cell lines. Cells were stimulated with 1 ng/mL of TGF‐β1 for 18 h, and luciferase activity was measured. (B) Ectopic Smad3 expression in HepG2 cells has different effects on the activity of CAGA_12_‐MLP‐Luc and SSS‐Luc. Smad3 was transiently expressed. Luciferase activity is expressed as fold induction by TGF‐β. Error bars represent the *SD* from three technical replicates.

To examine whether the preference of Smad complexes for SBE or CAGA motifs in different cells affects transcriptional activity, we used the luciferase reporters CAGA_12_‐MLP‐Luc^27^ and SSS‐Luc,^16^ which are both composed only of Smad‐binding motifs, CAGA or SBE, and are devoid of cofactor‐binding motifs. These reporters are thus driven principally by Smad proteins without the aid of Smad cofactors. The activity of these reporters was determined in the three cell lines as well as in NMuMG normal murine mammary gland epithelial cells (Figure 1A). SSS‐Luc, an SBE‐based reporter, exhibited higher activity than CAGA_12_‐MLP‐Luc in HepG2 cells, but not in other cell lines. The results are consistent with the above observation that activated Smad complexes in HepG2 cells show a lower preference for sequences containing CAGA motifs. Thus, the activated Smad complexes themselves exhibit cell type‐specific differences in their capacity to activate SBE or CAGA‐based reporters.

Because HepG2 cells express low levels of the Smad3 protein (Ref. [13] and Figure S2), activated Smad complexes in HepG2 cells may have a lower amount of the Smad3 protein preferring CAGA motifs to SBE. To address this possibility, Smad3 was ectopically expressed in HepG2 cells and reporter activity was measured. The results showed that the fold induction of CAGA_12_‐MLP‐Luc activity by TGF‐β was higher than that of SSS‐Luc (Figure 1B), suggesting that the relative activity of artificial reporters is affected by the expression levels of Smad proteins in target cells.

### 3.3 Transcription factor‐binding motifs co‐enriched with SBE or CAGA motifs

**FIGURE 2:**
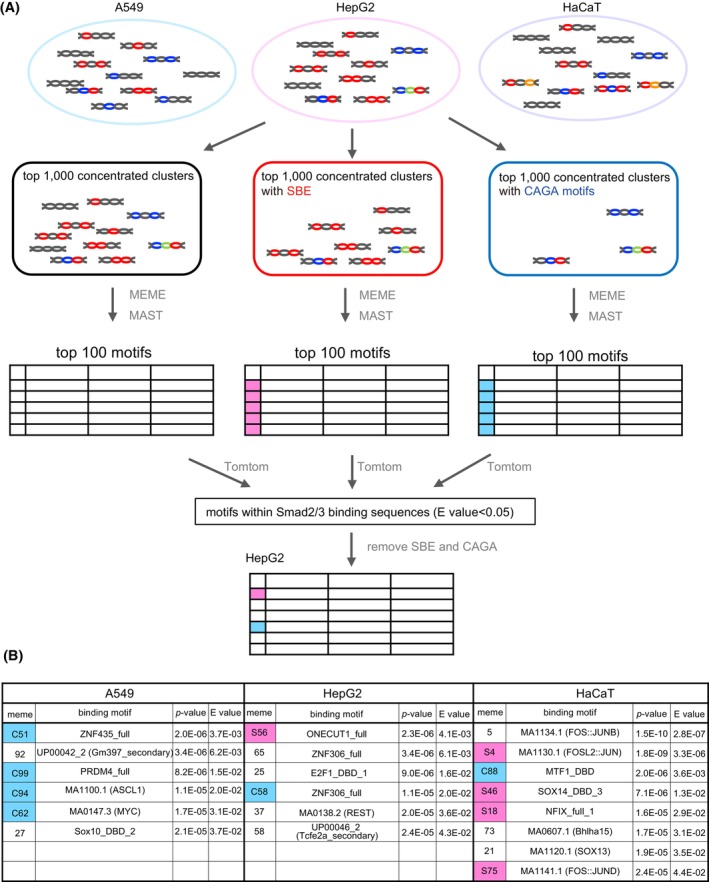
Transcription factor‐binding motifs concentrated in Smad2/3‐binding oligonucleotides. (A) A scheme for the identification of transcription factor‐binding motifs. Concentrated nucleotides were divided into three groups, clusters containing SBE or CAGA, and nonselected clusters. These clusters were analyzed by MEME and MAST to detect target motifs. The target motifs were compared with known transcription factor‐binding motifs by Tomtom. (B) Predicted transcription factor‐binding motifs are listed. MEME motifs obtained from nonselected clusters and clusters containing SBE (S) or CAGA (C) are indicated together with *p*‐values and *E* values.

We next investigated whether the Smad complex‐binding sequences contain binding motifs for transcription factors other than Smad (Figure 2). The top 1000 clustered sequences in each cell line were analyzed using MEME (multiple Em for Motif Elimination, https://meme‐suite.org/meme/tools/meme)^18^ and MAST (Motif Alignment & Search Tool, https://meme‐suite.org/meme/tools/mast).^19^ To determine whether SBE or CAGA motifs co‐concentrate with specific transcription factor‐binding motifs, clustered sequences were also divided into two groups, those containing SBE and those containing CAGA motifs, and similarly subjected to analysis using MEME and MAST. The 100 motifs identified by MEME were compared with motifs from databases (JASPAR2018_CORE_vertebrates_nonredundant, uniprobe_mouse, and Ref. [28]) using Tomtom (Motif comparison Tool, https://meme‐suite.org/meme/tools/tomtom).^20^ The top predicted transcription factors from each motif matrix are listed in ascending order of *E*‐values in the Tomtom analysis in Table S2. MEME motifs that were redundant were merged, those with SBE or CAGA motifs were omitted, and those with *E* > .05 were also omitted from the list, leaving 20 MEME motifs (Figures 2 and S3). The list contained predicted motifs for previously reported Smad cofactors, namely, Fos/Jun (MEME motifs: HaCaT‐5, HaCaT‐S4, and HaCaT‐S75)^29^, ^30^ and Sox (MEME motifs: HaCaT‐21, HaCaT‐S46, and A549‐27),^31^ thus validating the selection procedure.

### 3.4 Construction of reporter plasmids composed of the Fos/Jun family binding motifs and SBE

**FIGURE 3:**
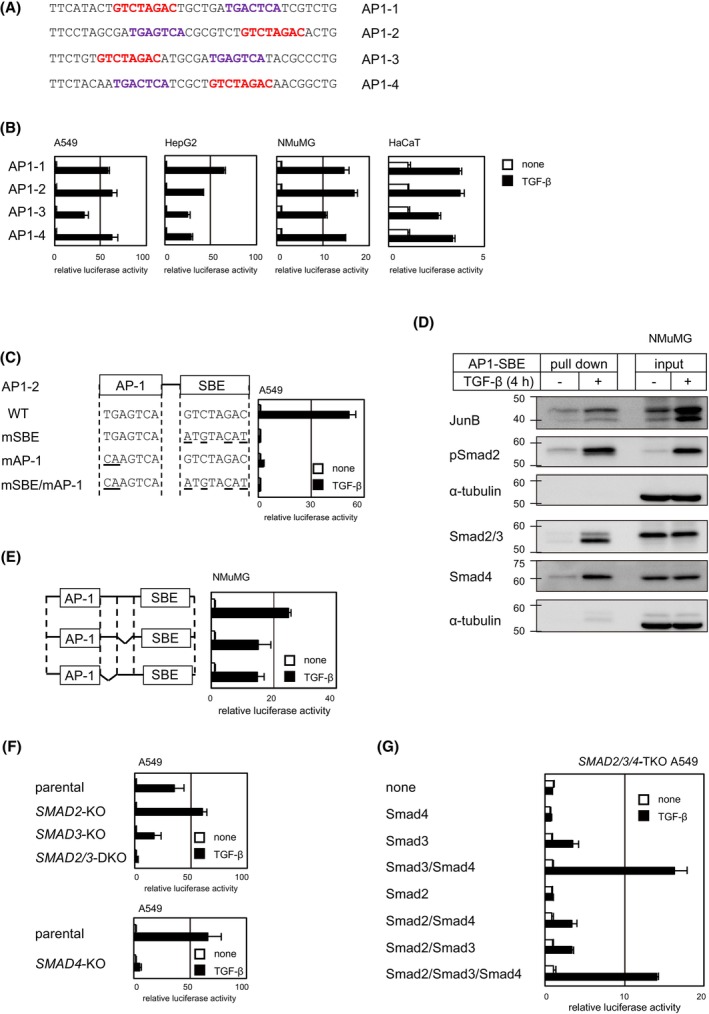
TGF‐β‐induced activation of reporters composed of the AP‐1‐binding motif and SBE. (A) Selected sequences for constructing reporters. The AP‐1‐binding motif and SBE are colored *purple* and *red*, respectively. (B) Luciferase reporter activity in response to TGF‐β stimulation in A549, HepG2, NMuMG, and HaCaT cells. (C) Mutations in the AP‐1 motif and/or the SBE attenuated TGF‐β‐induced reporter activity. Mutated residues are underlined. (D) DNA affinity precipitation assay using biotinylated AP1‐SBE oligonucleotide as a probe. Cell lysates from NMuMG cells with or without TGF‐β stimulation (4 h) were subjected to analysis. Binding of JunB and Smad proteins to the AP1‐SBE oligonucleotide was detected by immunoblotting. α‐tubulin was used as a loading control. (E) Deletion of three nucleotides from the linker sequence between the AP‐1 motif and the SBE (seven nucleotides) attenuated reporter activity. (F) Activation of AP1‐SBE‐Luc by TGF‐β in parental, *SMAD2*‐KO, *SMAD3*‐KO, *SMAD2/3*‐DKO, or *SMAD4*‐KO A549 cells. (G) Activation of AP1‐SBE‐Luc by TGF‐β in *SMAD2/3*/*4*‐triple KO A549 cells rescued by transient expression of Smad2, Smad3, and/or Smad4. Luciferase activity is expressed as fold induction by TGF‐β (B, C, E, and F) or relative values to those in non‐transfected cells without TGF‐β stimulation (G). Error bars represent the *SD* from three experimental replicates.

We next inspected sequences containing the Fos/Jun‐binding motif (activator protein‐1 [AP‐1] binding motif, 5′‐TGA(G/C)TCA‐3′). Concentrated sequences with the MEME motif HaCaT‐S4 (5′‐GNRTGASTCATC‐3′) and HaCaT‐5 (5′‐RTGACTCAT‐3′), where R denotes G/A and S denotes G/C, are listed in Figure S4A. The AP‐1‐binding motif was mostly co‐enriched with SBE, but not with CAGA motifs. The distances between the AP‐1‐binding motif and SBE ranged within five to nine bases, suggesting that a specific distance is required for Smad2/3 binding to DNA. We arbitrarily selected four sequences and inserted them into the pGL4‐MLP plasmid for constructing reporters (Figure 3A). All four reporters were activated by TGF‐β in A549, HepG2, NMuMG, and HaCaT cells (Figure 3B). Although the sequences were obtained by CASTing analysis of HaCaT cells, fold induction was not high in HaCaT cells; the reporter activities were higher in other cell lines.

We further characterized the reporter construct with the sequence AP1‐2. Reporter activation was attenuated by introducing a mutation in the AP‐1 motif or the SBE, indicating that both the AP‐1 motif and the SBE are necessary for the induction of reporter activity by TGF‐β (Figure 3C). Deletion of the upstream AP‐1 flanking sequence (5′‐CTAG‐3′) increased the TGF‐β response, whereas further deletion did not affect the response (Figure S4B). Thereafter, the reporter with a 4‐base deletion was used for further characterization (AP1‐2m4‐D2, thereafter termed AP1‐SBE‐Luc). A DNA affinity precipitation assay indicated that Smad2/3, Smad4, and JunB bound to the AP1‐SBE probe in response to TGF‐β stimulation (Figure 3D). We previously reported that a single SBE is not sufficient for binding to the activated Smad complex.^16^ The AP1‐SBE probe contains a single SBE, but successfully interacted with the Smad complex, suggesting cooperative binding with JunB or other Jun family proteins.

Deletion of three nucleotides from the linker sequence between the AP‐1 motif and the SBE resulted in attenuated reporter activity (Figure 3E), suggesting that the distance between two motifs is important. Therefore, transcription factors that bind to the AP‐1 motif or the SBE may cooperatively, but not independently, activate transcription. Substitution of the SBE with other Smad‐binding motifs, CAGA motifs, abrogated reporter activity (Figure S4C). However, the reporter activity was maintained when the SBE was substituted with a triple‐CAGA motif (Figure S4D). These results are consistent with previous data from our group showing that a single SBE is interchangeable with a triple‐CAGA motif in inducing Smad‐dependent transcriptional activation.^16^ The reason for the exclusive co‐concentration of the AP‐1 motif with SBE may be the length limitation of random nucleotide sequences in the oligonucleotide library.

TGF‐β‐induced reporter activity was enhanced in *SMAD2*‐knockout cells, attenuated in *SMAD3*‐knockout cells, and almost abrogated in *SMAD2/3*‐double knockout cells (Figure 3F, top), indicating that Smad3 plays a central role in the transcriptional activation of the reporter, although Smad2 also plays a role in the absence of Smad3. There was some residual activity induced by TGF‐β in *SMAD4*‐knockout cells but not in *SMAD2/3/4*‐triple knockout cells (Figure 3F, bottom, and Figure 3G), suggesting that Smad complexes devoid of Smad4 can activate this reporter to some extent. To verify the dependency of the reporter on Smad proteins, we performed rescue experiments in *SMAD2/3/4*‐triple knockout cells. Reporter activity was moderately rescued by expression of Smad3 alone or co‐expression of Smad2 and Smad4, but most efficiently by co‐expression of Smad3 and Smad4 (Figure 3G). This suggests that the reporter can be activated by Smad3 alone or a complex of Smad2 and Smad4, and most efficiently by a complex of Smad3 and Smad4.

### 3.5 Reporters with the Fos/JunD family‐binding AP‐1 motif exhibit distinct properties

**FIGURE 4:**
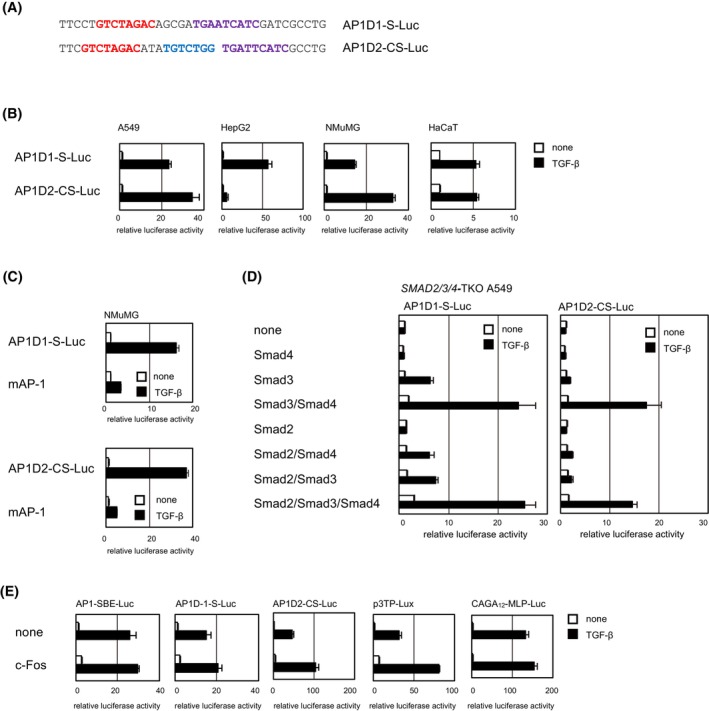
TGF‐β‐induced activation of reporters composed of the Fos/JunD‐binding AP‐1 motif and SBE. (A) Selected sequences for constructing reporters. The AP‐1‐binding motif, CAGA and SBE are colored *purple*, *blue* and *red*, respectively. (B) Luciferase reporter activity in response to TGF‐β stimulation in A549, HepG2, NMuMG, and HaCaT cells. (C) Mutations in the AP‐1 motif attenuated TGF‐β‐induced reporter activity in NMuMG cells. (D) Activation of composite reporters by TGF‐β in *SMAD2/3*/*4*‐triple KO A549 cells rescued by transient expression of Smad2, Smad3, and/or Smad4. (E) Effects of c‐Fos overexpression on reporter activity in NMuMG cells. c‐Fos was transiently expressed. Luciferase activity is expressed as fold induction by TGF‐β (B, C, and E) or relative values to those in non‐transfected cells without TGF‐β stimulation (D). Error bars represent the *SD* from three experimental replicates.

We next selected two sequences from the MEME‐motif HaCaT‐S75 (5′‐TGADTCATC‐3′, D = G/A/T), a predicted transcription factor of which is Fos/JunD, and constructed reporters (Figure 4A). One reporter contained the Fos/JunD motif (5′‐TGAATCA‐3′) and SBE (AP1D1‐S‐Luc), whereas the other one contained the Fos/JunD motif (5′‐TGATTCA‐3′), CAGA, and SBE (AP1D2‐CS‐Luc). These reporters responded to TGF‐β in all four cell lines (Figure 4B). Mutation of the Fos/JunD‐binding AP‐1 motif in these reporters repressed their activity, verifying the requirement of the AP‐1 motif for TGF‐β‐induced reporter activity (Figure 4C). TGF‐β induced the AP1D1‐S‐Luc reporter activity more efficiently than that of AP1D2‐CS‐Luc in HepG2 cells, whereas the opposite trend was observed in A549 and NMuMG cells. These results indicate that AP1D1‐S‐Luc and AP1D2‐CS‐Luc possess distinct properties. One possibility is that the difference in reporter activity reflects differences in the composition of the complexes containing transcription factors including Smad proteins.

We thus examined the dependency of reporter activities on Smad proteins (Figure 4D). AP1D1‐S‐Luc exhibited dependency on Smad proteins similar to that of AP1‐SBE‐Luc: The reporter activity was weakly rescued by Smad3 alone or the combination of Smad2/Smad4, but efficiently by the combination of Smad3/Smad4. However, AP1D2‐CS‐Luc was rescued by Smad3/Smad4, but only marginally by Smad3 alone or Smad2/Smad4. We further examined the effects of Fos/Jun overexpression on reporter activities. Overexpression of c‐Jun, JunB, or JunD inhibited TGF‐β–induced AP1‐SBE‐Luc and CAGA_12_‐MLP‐Luc reporter activities (Figure S5), consistent with a previous report using SBE4‐Lux.^32^ Therefore, it is difficult to evaluate the effect of Jun family proteins on AP‐1/SBE based reporters. By contrast, overexpression of c‐Fos weakly enhanced CAGA_12_‐MLP‐Luc activity (Figure 4E). AP1‐SBE‐Luc and AP1D1‐S‐Luc were weakly enhanced, whereas AP1D2‐CS‐Luc activity was more than doubled by c‐Fos overexpression (Figure 4E). In this experiment, p3TP‐Lux was used as a control for the AP‐1 motif‐containing TGF‐β‐responsive reporter.^33^ Similar to AP1D2‐CS‐Luc, p3TP‐Lux activity was more than doubled by c‐Fos overexpression. These results suggest that there are at least two subtypes of TGF‐β‐responsive reporters containing the AP‐1 motif.

### 3.6 Reporter assays identify novel candidate Smad cofactors

We next constructed reporters using concentrated sequences containing other 17 putative transcription factor‐binding motifs identified by MEME. We arbitrarily selected one of these sequences containing each MEME motif for construction (Figure S6 and Table 1), avoiding sequences with two SBEs because such sequences could respond to TGF‐β without the aid of Smad cofactors.^16^

**FIGURE 5:**
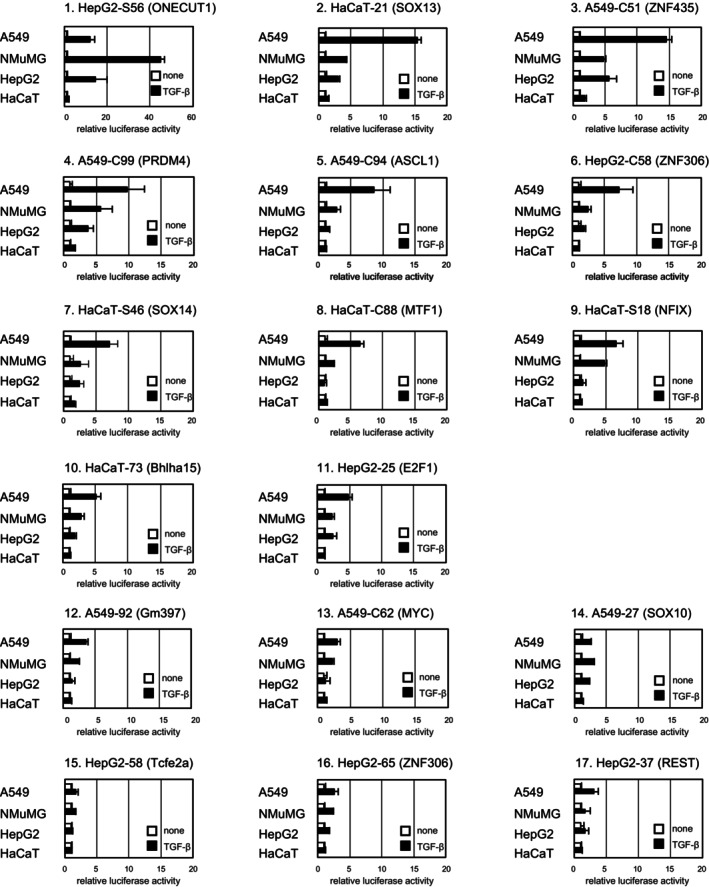
TGF‐β‐induced activation of reporters composed of putative transcription factor‐binding motifs and Smad‐binding motifs. Luciferase reporter activity in response to TGF‐β stimulation in A549, NMuMG, HepG2, and HaCaT cells. Data are shown as fold induction by TGF‐β. Error bars represent the *SD* from three experimental replicates. Candidate transcription factors for binding motifs in each reporter are shown in parentheses.

We then measured the reporter activities induced by TGF‐β in four cell lines, A549, HepG2, HaCaT, and NMuMG cells (Figure 5). Among the reporters with 17 motifs, those with 11 motifs exhibited more than fivefold activation by TGF‐β in at least in one of the four cell lines tested, whereas those with six motifs hardly responded to TGF‐β stimulation (less than fourfold activation) in all cell lines.

**FIGURE 6:**
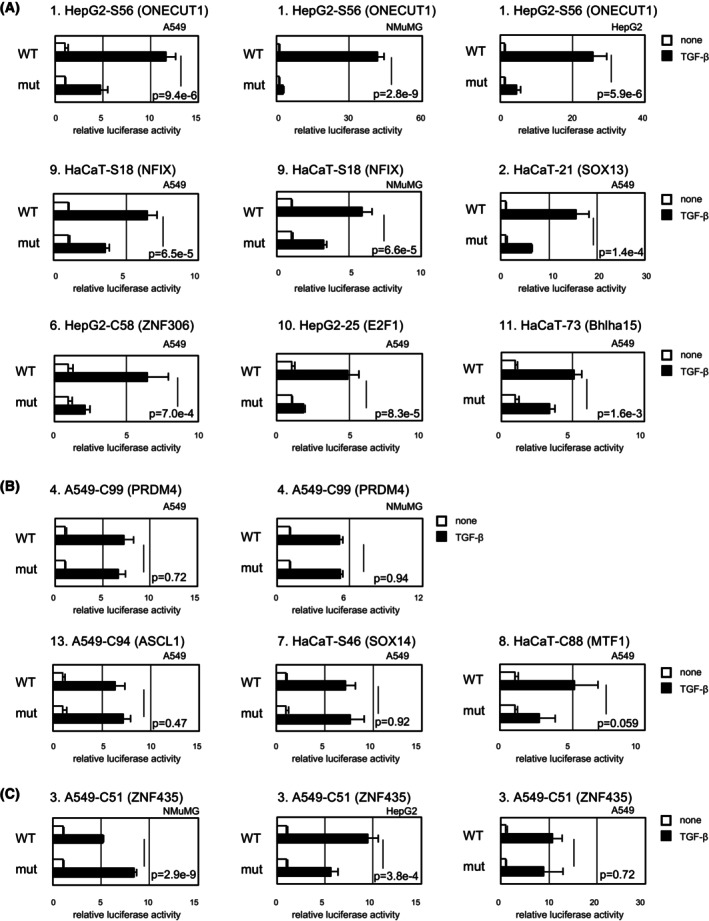
Requirements of putative transcription factor‐binding motifs for TGF‐β‐induced activation of reporter constructs. Activities of reporters with mutations in transcription factor‐binding motifs in response to TGF‐β stimulation. (A) Reporter activities attenuated by mutations. (B) Reporter activities not affected by mutations. (C) Reporter activities differently affected by mutations in three cell lines. Data are shown as fold induction by TGF‐β. Error bars represent the *SD* from three experimental replicates. Candidate transcription factors for binding motifs in each reporter are shown in parentheses. The data were subjected to statistical analysis using the Tukey's multiple comparison test. *p* < .05 indicated statistical significance.

Mutations in MEME motifs were then introduced in the 11 reporters activated by TGF‐β by referring to previous reports on candidate transcription factors if available or simply by replacing motifs with consecutive adenine residues (Table 1). The introduction of mutations significantly attenuated the activity of six reporters (Figure 6A), indicating that these MEME‐presented motifs are involved in transcriptional activation, whereas it did not affect the activity of four reporters (Figure 6B), indicating a minimum contribution of the MEME motifs to transcriptional activation. The former motifs could be responsive elements to Smad cofactors. These candidate Smad cofactors include Onecut1, NFIX, SOX13, E2F1, ZNF306, and Bhlha15.

One reporter (A549‐C51) was highly activated in NMuMG cells by a mutation in a putative transcription factor‐binding motif (Figure 6C). This motif might play a negative role in TGF‐β‐induced transcription in NMuMG cells, and other parts of the reporter sequences may contribute to transcription. However, its role was cell‐type dependent: the mutation attenuated the activation of the reporter in HepG2 cells or did not affect it in A549 cells.

## 4 DISCUSSION

TGF‐β is a cytokine that is widely distributed throughout the body and its receptors are also ubiquitously expressed. The mechanism by which TGF‐β regulates gene expression in a context‐dependent manner to evoke distinct responses in different target cells is one of the long‐standing questions in the field. In this study, we aimed to elucidate the molecular basis of context‐dependent cell signaling by analyzing the distinct DNA‐binding properties of activated Smad complexes. To this end, we used the CASTing analysis to concentrate DNA sequences that interact with endogenous Smad complexes isolated from cells stimulated with TGF‐β using an anti‐Smad2/3 antibody.

We first observed that the activated Smad complexes interacted with DNA sequences containing CAGA motifs less efficiently in HepG2 than in A549 and HaCaT cells (Table 2). Consistently, SSS‐Luc, an SBE‐based reporter, was more active than the CAGA_12_‐MLP‐Luc reporter in HepG2 cells, whereas CAGA_12_‐MLP‐Luc was more active in HaCaT, A549, and NMuMG cells (Figure 1A). One explanation for this result is the low expression of Smad3 in HepG2 cells, because ectopic expression of Smad3 altered the trend (Figure 1B). The balance of Smad2 and Smad3 affects the response to TGF‐β signaling,^9^, ^12^ and differences in the composition of Smad transcriptional complexes may contribute to context‐dependent transcription, as previously predicted using a bioinformatics approach.^13^ Here, we experimentally demonstrated this possibility, although the nature of the different activated Smad complexes remains to be elucidated in detail.

Different expression/activation profiles of Smad cofactors are also involved in context‐dependent transcription.^34^ Recently, ChIP‐seq or ChIP‐chip data from various cell lines stimulated with TGF‐β have been accumulated, and candidate Smad cofactors can thus be found by searching for transcription factor‐binding motifs in Smad2/3‐binding genomic DNA sequences. Koinuma et al.^21^ found that AP‐1, AP‐2, and ETS motifs are enriched in Smad2/3‐binding regions, in addition to the Smad‐binding 5′‐AGAC‐3′ motif, in HaCaT cells stimulated with TGF‐β. Mizutani et al.^22^ reported that HNF‐4, AhR‐ARNT, and n‐Myc motifs are enriched together with a Smad‐binding 5′‐CAGAC‐3′ motif. However, such approach may be biased by motifs that are frequently found in the genome. In this study, we showed that Smad proteins interact with SBE. However, SBE is not frequently detected even in the Smad2/3‐binding genomic region.^16^ In addition, Smad cofactors that regulate a smaller subset of target genes may be overlooked. We examined the occurrence of binding motifs mediating interactions with candidate Smad cofactors in Smad2/3‐binding genomic sequences obtained by ChIP‐seq (A549, GSE51510) or ChIP‐chip (HaCaT, GSE11710 and HepG2, and GSE28798) (within ±500 bp from the peak positions, thereafter referred as S2/3‐ChIP sequences) using FIMO (Find Individual Motif Occurrences, https://meme‐suite.org/meme/tools/fimo)^25^ (Table S3). As shown in Figure 6A, ONECUT transcription factors^35^ are good candidate Smad cofactors (MEME motif: HepG2‐S56). We recently verified that Onecut1 and Onecut2 can physically interact with Smad3, and overexpression of Onecut2 enhanced, whereas knockdown of Onecut2 attenuated the reporter activity (H. Fu and K. Miyazawa, unpublished observation). However, their occurrence was as low as 10% in the three cell lines examined, with *q*‐values of 1, indicating that they were not significantly concentrated. These results support the usefulness of the current method for identifying novel Smad cofactors.

The cooperation of Fos/Jun proteins with Smad proteins has been known since the early days after identification of the Smad signaling pathway.^29^, ^36^, ^37^ However, the underlying mechanism is not well understood. Some reports suggest that Smad and AP‐1 proteins independently bind to DNA and cooperate in transcriptional activation,^32^, ^36^ whereas others indicate that Smad proteins interact with the AP‐1 motif^29^ or AP‐1 proteins interact with SBE.^38^ To address this point, we performed a DNA affinity precipitation assay and found that both Smad and AP‐1 proteins interact with the AP1‐SBE DNA probe. Substitution of the AP‐1 motif or the SBE in AP1‐SBE‐Luc with the other attenuated reporter activity (Figure S4E), suggesting that the binding specificity of each element is not promiscuous, and binding of both proteins is required for transcriptional activation. We also found that there are at least two subtypes of TGF‐β‐responsive reporters containing the AP‐1 motif and that they have distinct Smad dependencies and sensitivities to c‐Fos overexpression. p3TP‐Lux, first reported in 1992, consists of three repeats of the AP‐1 motif derived from the human collagenase gene, together with the −740/−636 region of the PAI‐1 promoter containing three CAGA motifs.^33^ We newly developed reporters with properties distinct from that of p3TP‐Lux.

The CASTing system can enrich DNA sequences that are preferentially bound by activated Smad complexes. We successfully established artificial reporters using some of the enriched sequences, as shown in Figures 3, 4, 5, but not using others. Therefore, this assay system can enrich sequences that are preferred by complexes of Smad and Smad‐binding transcription factors, but these may not always be suitable for transcriptional activation. We constructed reporters using three sequences from HepG2‐S56 MEME cluster, representing ONECUT‐binding motif, but only one of them exhibited a good response and other two sequences did not respond at all to TGF‐β stimulation (Figure S6 and unpublished observations). For each candidate Smad cofactor other than AP‐1 and Onecut proteins, we tested only one sequence for reporter activity, and testing additional sequences may be necessary to identify those that respond well to TGF‐β.

Although we detected Fos/Jun and Sox in our screening, binding sequences for other well‐known cofactors including FoxO,^39^ p53,^40^, ^41^ Gli,^42^ and TEAD^43^ were not significantly concentrated. Gli2 and TEAD were found in the early stages of our screening (Table S2), but they were dropped in the later stages (Figure 2). Therefore, the current protocol is not sufficient for comprehensively detecting Smad cofactors. This may be because we used nuclear extracts obtained from cells stimulated for 1 h by TGF‐β. Smad cofactors that are induced or activated after TGF‐β stimulation may not be included. For example, the AP‐1 motifs were enriched in Smad‐binding sequences obtained from HaCaT cells, but not other cell lines. However, the reporter construct was active in all three cell lines. Alternatively, transcription factors with longer consensus sequences such as p53 might be excluded because of the limitation in the length of the random nucleotide region. In pilot experiments, the use of shorter random sequences of 12 or 18 nucleotides in the CASTing analysis resulted mostly in concentrated sequences containing 5′‐GTCTAGACGTCT‐3′ or 5′‐GTCTAGACXXXTGTCTGG‐3′, respectively. The use of these shorter sequences would prevent us from detecting putative Smad cofactor‐binding sequences. Therefore, the length of random nucleotide regions affects the profiles of detected Smad cofactors.

As described above, this assay system is not sufficient to comprehensively detect candidate Smad cofactors. Nevertheless, we were able to identify several novel candidate Smad cofactors. Altering the length of random nucleotide sequences or the time point for harvesting nuclear extracts after TGF‐β stimulation may enable the detection of additional candidates as Smad cofactors. The identification of novel Smad cofactors, together with examining the histone modification status of target genes, would contribute to our understanding of the role of context‐dependent TGF‐β signaling in health and disease.

## AUTHOR CONTRIBUTIONS

Yuka Itoh, Kunio Miyake, and Keiji Miyazawa acquired and analyzed CASTing data. Yuka Itoh and Chiho Omata performed most of other experiments. Yuka Itoh, Masao Saitoh, and Keiji Miyazawa designed other experiments and analyzed data. Yuka Itoh and Daizo Koinuma analyzed ChIP‐seq data. Yuka Itoh, Masao Saitoh, and Keiji Miyazawa wrote the manuscript. All the authors reviewed the manuscript and provided approval for submission.

## FUNDING INFORMATION

This work was supported by the Japan Society for the Promotion of Science (JSPS) KAKENHI [16H05150, 18K06626, 19KK0400, 21H02762, and 21K06527], and the Fugaku Trust for Medicinal Research, the Mitsubishi Science Foundation, and the Terumo Foundation for Life Science and Arts.

## DISCLOSURES

The authors declare that they have no conflicts of interest related to the content of this article to disclose.

## Supporting information


Data S1.

## Data Availability

Raw data of CASTing analysis have been deposited with links to BioProject identifier PRJDB9205 in the DDBJ BioProject database (https://ddbj.nig.ac.jp/search/entry/bioproject/PRJDB9205). Other original data are contained within the article and its Supporting Information.
