# Supplementary material for: Analysis of the DNA‐binding properties of TGF‐β‐activated Smad complexes unveils a possible molecular basis for cellular context‐dependent signaling
Source: FASEB J. 2024 Aug 8;38(15):e23877. doi: 10.1096/fj.202400978R (PMC11607640; doi:10.1096/fj.202400978R)
Supplement: Supplementary file 1 — Data S1. [file FSB2-38-e23877-s001.pdf]

Supporting information for

Analysis of the DNA-binding properties of TGF-β–activated Smad complexes unveils a possible molecular basis for cellular context-dependent signaling

Yuka Itoh, Kunio Miyake, Daizo Koinuma, Chiho Omata, Masao Saitoh, and Keiji Miyazawa

Supplementary Tables: Table S1-S3

Table S1. Top 30 concentrated sequences from the CASTing analysis of Smad2/3 in A549, HepG2, and HaCaT cells stimulated with TGF-β

Table S2. Top 25 predicted transcription factor-binding motifs from MEME, MAST, and Tomtom analyses of Smad2/3-binding sequences obtained by CASTing analysis

Table S3. Occurrence of the MEME motifs in Smad2/3-binding sites obtained by ChIP-seq/chip analyses

Supplementary Figures: Figure S1-S6

Figure S1. Establishment of *SMAD2/3/4*-triple knockout A549 cells

Figure S2. Expression of Smad2, Smad3 and Smad4 in A549, HepG2, HaCaT and NMuMG cells

Figure S3. Transcription factor-binding motifs concentrated in Smad2/3-binding sequences obtained by CASTing analysis

Figure S4. Transcriptional activation of the AP-1 binding motif and SBE composite reporters in response to TGF-β stimulation

Figure S5. Effects of overexpression of Jun family proteins on AP1-SBE-Luc or $CAGA_{12}$-MLP-Luc activities in response to TGF-β stimulation

Figure S6. Concentrated sequences containing 17 selected motifs presented by MEME

Table S1.

Top 30 concentrated sequences from the CASTing analysis of Smad2/3 in A549, HepG2, and HaCaT cells stimulated with TGF-β

	A549	HepG2	HaCaT
1	TCGCCGAATGTCTAGACATATGTCTGGTTCGTCT	TCCAATGTGTCTAGACACCGTCTAGACGGGCGCT	TCGGGCATATGTCTAGACACTGTCTGGTTTGCCT
2	TCGTGTGTAACTCGCTAGTCGGGGAGATAATTCT	TCGTTACGTCTAGACATAGTCTAGACAGCTTGCT	TCACGCACGCAGGCCCCGCCAATTCTGTCTAGCT
3	TCGAAGATATATAGTGGCTGGCGGCAGTTTAACT	TCGTAGCGGGATGTCTAGACGTCTAGACGGCGCT	TCGATGGCCTGTCCAGACAGTGTCTAGACACCCT
4	TCGCACTGTCTAGACACTGTCTAGACGCGTTGCT	TCGAATATCCCGTCTAGACGGGTGTCTAGACACT	TCGTCATGCTTGACTGCAACTGTCTAGACGCCCT
5	TCGCATGTCTAGACACTGTCTGGTTACTCTGGCT	TCGCGTGTGTCTAGACAGGTGTCTAGACCGTGCT	TCGATTGTCTAGACAGTGTCTGGAATCCTATCCT
6	TCCACCGTGTTACTGTCTAGACAGTGTCTGGCCT	TCGCCGTGTCTAGACGATTGTCTAGACGTTCCCT	TCGTGATGTCCGCTGTCTAGACAGTGTCTAGACT
7	TCGCGTGTCTAGACACTGTCTGGGTTCCCGGGCT	TCGGCCGTAACGTGTCTAGACGTCTAGACGGGCT	TCGCAATGTCTAGACACTGTCTGGGTCGCACCCT
8	TCCAGACACTGTCTAGACATGTAGTGGCTGGGCT	TCGTGTGTCTAGACAATCGTCTAGACGCGTTGCT	TCATTGTGTCTAGACAGTGTCTAGACAGTGCGCT
9	TCGTCAGCCTGTGCGGTCAGTCGGAGAGCATTCT	TCGTCGTCTAGACAGTGTCTAGACCGCACGCGCT	TCGGGAGCATGCGGGGTCGGCAGTCGGAGAGACT
10	TCCTGTCTAGACACTGTCTGGAATTTGCTGCGCT	TCGACACGTCTGGTGTCTAGACTTGGTCTAGACT	TCGTTCCAGACATTGTCTAGACGGGTGTCTCGCT
11	TCGTTGCGGACGTAGTCTAGACACTGTCTAGACT	TCGTAAATGTCTAGACGTCTAGACACGCCACCCT	TCGTCTGTAAACTGTCTAGACAACTGTCTAGACT
12	TCGTCTGACTGTCTAGACATGCTGTCTGGATCCT	TCGCGTAGGTTGTCTAGACAGTGTGTCTAGACCT	TCCACACTGTCTAGACTGTCTGTCTAGACGCCCT
13	TCGTGTCTAGACATTGTCTGGAATTGTGGCGGCT	TCAGTGTCTAGACGGTGTCTAGACAGCCCCGGCT	TCGCAACTGTCTAGACATATGTCTAGCTGGGCCT
14	TCCAGACAGTGTCTAGACAGCAAACGAGGGGCCT	TCGTAGCGGGATGTCTAGACGTCTAGAGGCGCT	TCCGCTTGACTGGCGACGTCTAGACATGTGCCCT
15	TCGGAACGCCAACTCGCCGGTCGGGGAGCGCTCT	TCGAACTATGTCAGTCTAGACGTCTAGACAGGCT	TCCAGACACTGTCTAGACATCGGCGGGAGGTCCT
16	TCGTGTGCAACTCGCTGGTCGGAGAGACAAATCT	TCGGCTGGGTGAGTCTAGACATGGTCTAGACGCT	TCGGTTGGTCTAGACATATGTCTAGACACAGCCT
17	TCGAGGGTTATGTCTAGACAGTATGTCTGGCACT	TCGGTGTTGGGTCTAGACACCTGTCTAGACGACT	TCCAGACACTGTCTAGACAGCTCCTGAGGCGACT
18	TCCAGACATTGTCTAGACACGCAGGAGACTGTCT	TCGTATGGGCTGTCTAGACGTCTAGACAGCGGCT	TCGCCTGTACGGATTTCCAGACACTGTCTAGACT
19	TCGTATGTCTAGACATGGTGTCTGGAATGTGGCT	TCGGTAGACTACTGTCTAGACAGCGTCTGGGCCT	TCGTGTCTAGACAGTGTCTGGCTTTGCGCAGCCT
20	TCGTGTGTAACTCGTCAGTCGGAGAGATAGAACT	TCCATGTCTAGACAACTGTCTAGACAAGATCCCT	TCAAAACATCGTGTCGCGCCTGTCGGGGAGTTCT
21	TCGTTGTGTCTAGACACTGTCTGGAAGCCACCCT	TCCAATGGCCCCCCGTCTAGACATTGTCTAGCCT	TCTGTCTAGACAGTGTCTGGATGTCTAGACCCCT
22	TCGGCATTGTCTAGACATGTGTCTGGGTCCGCCT	TCAAATCGATATGTCTAGACACACCGTCTGGGCT	TCGTCTAGACATATGTCTGGTTCCCGTCCCGTCT
23	TCAGCGGGGGAGTGACGCGCCTGTATAGAACACT	TCTCATGTCTAGACAGTCTGTCTAGACGGCGGCT	TCAGCGGGGGAGCGATGCGGCTGTTTAGCAATCT
24	TCCAGACAGTGTCTAGACTAGTGTGTCTGCTCCT	TCGTCTAGACATTGTCTAGACAGGAGGGAGGGCT	TCGTTGTCCCAGACACTGTCTAGACAGGGGTCCT
25	TCGAAGACGGTTGGGTCTAGACACTGTCTAGACT	TCGATGTCTAGACAGTGTCTGGCGGATACTGGCT	TCGAACACCAGACACTGTCTAGACATGCGCCGCT
26	TCGGTCTAGACACTGTCTAGTCTAATGTCTGGCT	TCGCCACTGTCTAGACAATGTCTGGAAGCTGCCT	TCGCATTCGTACTGTCTAGACAGTGTGTCTGGCT
27	TCGATCTGTCTAGACATGTGTCTGGGTCCGCTCT	TCGTAAGATGGACTGTCTAGACGCTGTCTGGACT	TCGTCTGTGACCTGTCTAGACACCTGTCTGGTCT
28	TCAACTAACGTTCTGTCTAGACACTGTCTAGACT	TCCTGGGGTGGCGTCTAGACACCGTCTAGACACT	TCCCAGACACTGTCTAGACACCCCATGTTGTGCT
29	TCGATGTCTAGACACTGTCTGGCCGCACCTTCT	TCGTCATACTGTCTAGACAGCTGTCTGGCCCGCT	TCGAATGTCTAGACATAGTGTCTGGCCCACTGCT
30	TCGCCCGTTTAACGTCTCGGAGAGAGTCTAGACT	TCCATGTCTAGACTGTGTCTAGACTAAGGGGGCT	TCGAATGTCTAGACACTGTCTGGCGGCCTACGCT

5′-TC-3′ and 5′-CT-3′ are common flanking sequences of the library. SBE (GTCTAGAC) and CAGA motifs (TGTCTGG) are colored *red* and *blue*, respectively.

Table S2.

Top 25 predicted transcription factor-binding motifs from MEME, MAST, and Tomtom analyses of Smad2/3-binding sequences obtained by CASTing analysis

A549

meme	binding motif	*p*-value	E value	meme	binding motif	*p*-value	E value	meme	binding motif	*p*-value	E value
1	SMAD3_DBD	3.7.E-08	6.7.E-05	C4	SMAD3_DBD	1.2.E-07	2.2.E-04	S19	SMAD3_DBD	1.6.E-06	2.9.E-03
4	SMAD3_DBD	4.6.E-07	8.4.E-04	C2	Tp73_DBD	1.0.E-06	1.9.E-03	S2	MA1153.1 (Smad4)	3.3.E-06	6.0.E-03
2	MA1153.1 (Smad4)	3.0.E-06	5.4.E-03	C51	ZNF435_full	2.0E-06	3.7E-03	S4	SMAD3_DBD	5.7.E-06	1.0.E-02
92	UP00042_2 (Gm397_secondary)	3.4.E-06	6.2.E-03	C99	PRDM4_full	8.2.E-06	1.5.E-02	S3	UP00000_1 (Smad3_primary)	6.8.E-06	1.2.E-02
27	Sox10_DBD_2	2.1.E-05	3.7.E-02	C1	SMAD3_DBD	8.3.E-06	1.5.E-02	S1	SMAD3_DBD	8.8.E-06	1.6.E-02
12	ZBTB49_DBD	6.5.E-05	1.2.E-01	C29	SMAD3_DBD	1.1.E-05	2.0.E-02	S51	ZNF524_full_2	6.2.E-05	1.1.E-01
9	ZBTB49_DBD	1.0.E-04	1.9.E-01	C94	MA1100.1 (ASCL1)	1.1.E-05	2.0.E-02	S100	MA0513.1 (SMAD2::SMAD3::SMAD4)	6.5.E-05	1.2.E-01
85	MYBL2_DBD_3	1.2.E-04	2.1.E-01	C62	MA0147.3 (MYC)	1.7.E-05	3.1.E-02	S47	UP00026_2 (Zscan4_secondary)	9.9.E-05	1.8.E-01
5	MA1100.1 (ASCL1)	1.2.E-04	2.2.E-01	C15	UP00042_2 (Gm397_secondary)	2.5.E-05	4.5.E-02	S26	MA0614.1 (Foxj2)	1.1.E-04	2.0.E-01
21	ZNF784_full	1.5.E-04	2.8.E-01	C5	MA0092.1 (Hand1::Tcf3)	2.7.E-05	4.8.E-02	S41	GLIS3_DBD	1.3.E-04	2.4.E-01
48	GLIS3_DBD	1.9.E-04	3.4.E-01	C16	UP00026_1 (Zscan4_primary)	3.0.E-05	5.4.E-02	S32	RUNX2_DBD_1	1.4.E-04	2.5.E-01
93	MA0509.1 (Rfx1)	2.0.E-04	3.7.E-01	C3	UP00000_1 (Smad3_primary)	7.6.E-05	1.4.E-01	S58	MA0006.1 (Ahr::Arnt)	1.6.E-04	2.9.E-01
28	NFIX_full_3	2.5.E-04	4.4.E-01	C7	MTF1_DBD	1.3.E-04	2.3.E-01	S91	CTCF_full	1.7.E-04	3.0.E-01
38	Nr2e1_DBD_1	2.6.E-04	4.7.E-01	C8	YY2_DBD	1.4.E-04	2.6.E-01	S46	MA1100.1 (ASCL1)	1.9.E-04	3.4.E-01
44	SMAD3_DBD	2.9.E-04	5.2.E-01	C80	MA0616.1 (Hes2)	1.8.E-04	3.2.E-01	S23	UP00026_1 (Zscan4_primary)	2.0.E-04	3.6.E-01
62	MA0507.1 (POU2F2)	3.2.E-04	5.8.E-01	C87	ETV6_full_2	1.8.E-04	3.2.E-01	S31	UP00074_2 (Isgf3g_secondary)	2.0.E-04	3.6.E-01
39	ZSCAN4_full	3.3.E-04	5.9.E-01	C32	E2F2_DBD_2	2.5.E-04	4.5.E-01	S61	MA0471.1 (E2F6)	2.1.E-04	3.8.E-01
34	RUNX3_full	3.8.E-04	6.8.E-01	C81	UP00184_1 (Lhx8_2247.2)	2.6.E-04	4.7.E-01	S93	UP00099_2 (Ascl2_secondary)	2.1.E-04	3.9.E-01
26	FOXO3_full_2	3.9.E-04	7.1.E-01	C13	UP00088_2 (Plagl1_secondary)	2.6.E-04	4.8.E-01	S60	ESRRG_full_2	2.6.E-04	4.7.E-01
99	MA1153.1 (Smad4)	4.1.E-04	7.5.E-01	C38	Hic1_DBD_1	2.9.E-04	5.2.E-01	S90	Hic1_DBD_1	2.9.E-04	5.2.E-01
96	CREB3L1_full_1	4.2.E-04	7.7.E-01	C98	HSFY2_DBD_1	3.7.E-04	6.7.E-01	S92	CEBPB_DBD	2.9.E-04	5.2.E-01
95	PRDM4_full	4.2.E-04	7.7.E-01	C24	UP00017_1 (Nkx3-1_primary)	3.8.E-04	6.9.E-01	S12	MA0138.2 (REST)	3.1.E-04	5.6.E-01
35	CENPB_full	4.3.E-04	7.7.E-01	C19	ZNF143_DBD	3.9.E-04	7.1.E-01	S22	RUNX3_full	3.2.E-04	5.8.E-01
46	MA0092.1 (Hand1::Tcf3)	4.4.E-04	7.9.E-01	C64	MA0520.1 (Stat6)	3.9.E-04	7.1.E-01	S34	EGR4_DBD_2	3.2.E-04	5.8.E-01
43	ZBTB7A_DBD	4.4.E-04	8.0.E-01	C30	UP00006_2 (Zic3_secondary)	4.2.E-04	7.7.E-01	S49	HOMEZ_DBD	3.2.E-04	5.9.E-01

HepG2

meme	binding motif	p-value	E value	meme	binding motif	*p*-value	E value	meme	binding motif	*p*-value	E value
1	SMAD3_DBD	2.5.E-09	4.5.E-06	C1	SMAD3_DBD	7.5.E-08	1.4.E-04	S1	SMAD3_DBD	3.8.E-10	6.8.E-07
3	MA1153.1 (Smad4)	2.4.E-07	4.3.E-04	C58	ZNF306_full	1.1.E-05	2.0.E-02	S4	MA1153.1 (Smad4)	7.7.E-09	1.4.E-05
4	SMAD3_DBD	5.5.E-07	9.9.E-04	C4	SMAD3_DBD	2.4.E-05	4.4.E-02	S2	SMAD3_DBD	1.5.E-06	2.7.E-03
2	SMAD3_DBD	1.3.E-06	2.3.E-03	C38	MA0508.2 (PRDM1)	3.7.E-05	6.7.E-02	S56	ONECUT1_full	2.3.E-06	4.1.E-03
65	ZNF306_full	3.4.E-06	6.1.E-03	C3	MA1153.1 (Smad4)	3.7.E-05	6.7.E-02	S3	MA1153.1 (Smad4)	7.5.E-06	1.3.E-02
25	E2F1_DBD_1	9.0.E-06	1.6.E-02	C17	UP00000_1 (Smad3_primary)	5.1.E-05	9.2.E-02	S31	MA0138.2 (REST)	2.0.E-05	3.6.E-02
32	SMAD3_DBD	1.1.E-05	2.0.E-02	C70	UP00026_2 (Zscan4_secondary)	5.9.E-05	1.1.E-01	S65	UP00003_1 (E2F3_primary)	3.3.E-05	6.0.E-02
34	SMAD3_DBD	1.1.E-05	2.0.E-02	C64	UP00020_2 (Atf1_secondary)	8.8.E-05	1.6.E-01	S82	ZBED1_DBD	3.7.E-05	6.7.E-02
37	MA0138.2 (REST)	2.0.E-05	3.6.E-02	C86	UP00000_1 (Smad3_primary)	9.4.E-05	1.7.E-01	S30	MA0516.1 (SP2)	4.8.E-05	8.7.E-02
58	UP00046_2 (Tcfe2a_secondary)	2.4.E-05	4.3.E-02	C54	UP00042_2 (Gm397_secondary)	1.0.E-04	1.9.E-01	S50	UP00046_2 (Tcfe2a_secondary)	4.8.E-05	8.7.E-02
100	YY2_full_2	9.6.E-05	1.7.E-01	C98	UP00052_2 (Osr2_secondary)	1.3.E-04	2.3.E-01	S49	YY2_full_2	7.9.E-05	1.4.E-01
23	ONECUT1_full	1.1.E-04	1.9.E-01	C49	SRF_DBD	1.3.E-04	2.3.E-01	S92	UP00227_1 (Duxl_1286.2)	1.0.E-04	1.9.E-01
86	YY2_full_2	1.1.E-04	2.0.E-01	C6	GLI2_DBD_1	1.3.E-04	2.4.E-01	S21	UP00000_1 (Smad3_primary)	1.4.E-04	2.5.E-01
92	SPDEF_full_1	1.2.E-04	2.1.E-01	C15	YY1_full	1.6.E-04	2.9.E-01	S69	FOXB1_DBD_1	1.8.E-04	3.3.E-01
55	UP00160_1 (Obox3_3439.1)	1.4.E-04	2.5.E-01	C72	UP00046_2 (Tcfe2a_secondary)	1.7.E-04	3.0.E-01	S76	MA0144.2 (STAT3)	1.9.E-04	3.4.E-01
57	ZNF306_full	1.4.E-04	2.6.E-01	C94	SOX8_DBD_1	1.8.E-04	3.2.E-01	S88	E2F3_DBD_2	2.1.E-04	3.8.E-01
56	RARG_DBD_3	1.8.E-04	3.2.E-01	C37	ZNF282_DBD	1.8.E-04	3.3.E-01	S32	HSFY2_DBD_1	2.1.E-04	3.8.E-01
31	MA0060.3 (NFYA)	2.3.E-04	4.2.E-01	C100	SCRT2_DBD	1.9.E-04	3.4.E-01	S15	UP00000_1 (Smad3_primary)	2.4.E-04	4.3.E-01
64	ZNF784_full	2.4.E-04	4.4.E-01	C48	UP00099_2 (Ascl2_secondary)	2.0.E-04	3.5.E-01	S48	ZNF784_full	2.4.E-04	4.4.E-01
11	VSX1_DBD	2.5.E-04	4.5.E-01	C19	UP00000_1 (Smad3_primary)	2.0.E-04	3.6.E-01	S27	SMAD3_DBD	2.9.E-04	5.2.E-01
7	HSF4_DBD	2.9.E-04	5.2.E-01	C47	UP00026_2 (Zscan4_secondary)	2.1.E-04	3.9.E-01	S95	MA0505.1 (Nr5a2)	3.0.E-04	5.4.E-01
27	SMAD3_DBD	2.9.E-04	5.2.E-01	C59	FOXB1_DBD_1	2.4.E-04	4.4.E-01	S80	GRHL1_DBD_2	3.0.E-04	5.5.E-01
70	ZNF238_DBD	3.2.E-04	5.8.E-01	C71	UP00042_2 (Gm397_secondary)	2.6.E-04	4.6.E-01	S98	UP00031_2 (Zbtb3_secondary)	3.3.E-04	6.0.E-01
63	AR_full	4.3.E-04	7.8.E-01	C55	RORA_DBD_2	3.0.E-04	5.4.E-01	S14	Ascl2_DBD	3.8.E-04	6.9.E-01
42	ZBED1_DBD	4.4.E-04	8.0.E-01	C56	ZNF435_full	3.1.E-04	5.5.E-01	S7	UP00008_2 (Six6_secondary)	3.9.E-04	7.0.E-01

HaCaT

meme	binding motif	p-value	E value	meme	binding motif	*p*-value	E value	meme	binding motif	*p*-value	E value
5	MA1134.1 (FOS::JUNB)	1.5.E-10	2.8.E-07	C5	SMAD3_DBD	1.0.E-08	1.8.E-05	S4	MA1130.1 (FOSL2::JUN)	1.8.E-09	3.3.E-06
2	SMAD3_DBD	8.9.E-07	1.6.E-03	C1	SMAD3_DBD	9.9.E-08	1.8.E-04	S2	SMAD3_DBD	1.3.E-07	2.3.E-04
1	SMAD3_DBD	5.3.E-06	9.6.E-03	C24	SMAD3_DBD	7.2.E-07	1.3.E-03	S3	MA1153.1 (Smad4)	2.5.E-06	4.6.E-03
30	SOX14_DBD_3	7.1.E-06	1.3.E-02	C3	MA1153.1 (Smad4)	1.5.E-06	2.8.E-03	S46	SOX14_DBD_3	7.1.E-06	1.3.E-02
73	MA0607.1 (Bhlha15)	1.7.E-05	3.1.E-02	C88	MTF1_DBD	2.0.E-06	3.6.E-03	S18	NFIX_full_1	1.6.E-05	2.9.E-02
21	MA1120.1 (SOX13)	1.9.E-05	3.5.E-02	C2	MA0513.1 (SMAD2::SMAD3::SMAD4)	2.0.E-05	3.6.E-02	S1	MA1153.1 (Smad4)	2.3.E-05	4.1.E-02
3	MA1153.1 (Smad4)	3.8.E-05	6.8.E-02	C52	MA0138.2 (REST)	5.0.E-05	9.0.E-02	S75	MA1141.1 (FOS::JUND)	2.4.E-05	4.4.E-02
51	NFE2_DBD	4.1.E-05	7.3.E-02	C42	SRY_DBD_3	8.1.E-05	1.5.E-01	S80	MA0477.1 (FOSL1)	3.4.E-05	6.2.E-02
93	ESRRA_DBD_5	6.0.E-05	1.1.E-01	C78	NKX2-3_full	8.8.E-05	1.6.E-01	S62	HINFP1_full_2	3.8.E-05	6.8.E-02
59	MA0506.1 (NRF1)	6.7.E-05	1.2.E-01	C49	UP00020_2 (Atf1_secondary)	8.8.E-05	1.6.E-01	S20	Hnf4a_DBD	4.6.E-05	8.2.E-02
84	HSF2_DBD	1.6.E-04	2.9.E-01	C4	UP00000_1 (Smad3_primary)	9.2.E-05	1.7.E-01	S39	TEAD3_DBD_2	8.6.E-05	1.6.E-01
12	MA0139.1 (CTCF)	1.8.E-04	3.3.E-01	C54	MA0092.1 (Hand1::Tcf3)	9.8.E-05	1.8.E-01	S82	UP00006_2 (Zic3_secondary)	1.1.E-04	2.0.E-01
52	UP00101_2 (Sox12_secondary)	2.3.E-04	4.1.E-01	C59	TEAD1_full_2	1.0.E-04	1.9.E-01	S90	UP00095_1 (Zfp691_primary)	1.4.E-04	2.5.E-01
56	UP00099_1 (Ascl2_primary)	2.3.E-04	4.2.E-01	C90	UP00054_1 (Tcf7_primary)	1.2.E-04	2.2.E-01	S70	YY2_full_2	1.4.E-04	2.5.E-01
88	GMEB2_DBD_3	2.4.E-04	4.3.E-01	C9	ONECUT1_full	1.4.E-04	2.5.E-01	S74	E2F3_DBD_2	1.5.E-04	2.7.E-01
55	SOX18_full_2	3.2.E-04	5.8.E-01	C60	ZNF524_full_1	1.7.E-04	3.1.E-01	S19	CUX1_DBD_1	2.3.E-04	4.2.E-01
45	UP00036_2 (Myf6_secondary)	3.2.E-04	5.9.E-01	C67	UP00015_2 (Ehf_secondary)	1.9.E-04	3.4.E-01	S65	Pou2f2_DBD_2	2.6.E-04	4.7.E-01
64	UP00048_1 (Rara_primary)	3.3.E-04	5.9.E-01	C100	MTF1_DBD	2.4.E-04	4.3.E-01	S52	NEUROD2_full	2.7.E-04	4.9.E-01
31	UP00000_1 (Smad3_primary)	3.4.E-04	6.1.E-01	C35	VDR_full	2.7.E-04	4.9.E-01	S26	UP00000_1 (Smad3_primary)	2.9.E-04	5.2.E-01
6	UP00102_2 (Zic1_secondary)	3.7.E-04	6.6.E-01	C82	UP00167_1 (En1_3123.2)	2.9.E-04	5.3.E-01	S50	UP00031_1 (Zbtb3_primary)	3.2.E-04	5.9.E-01
77	UP00068_2 (Eomes_secondary)	3.7.E-04	6.7.E-01	C58	ZSCAN4_full	3.0.E-04	5.4.E-01	S36	RARG_DBD_3	3.7.E-04	6.6.E-01
97	E2F2_DBD_2	3.7.E-04	6.8.E-01	C19	MA0258.2 (ESR2)	3.6.E-04	6.5.E-01	S61	RORA_DBD_1	3.9.E-04	7.0.E-01
100	ZBED1_DBD	4.3.E-04	7.8.E-01	C98	UP00060_2 (Max_secondary)	3.6.E-04	6.5.E-01	S16	MA0147.3 (MYC)	4.1.E-04	7.4.E-01
95	GLI2_DBD_1	4.6.E-04	8.3.E-01	C13	UP00095_1 (Zfp691_primary)	3.8.E-04	6.8.E-01	S27	RUNX3_full	4.2.E-04	7.5.E-01
8	UP00097_1 (Mtf1_primary)	4.7.E-04	8.4.E-01	C71	MA0597.1 (THAP1)	3.8.E-04	6.8.E-01	S72	NFIA_full_1	4.2.E-04	7.6.E-01

Motifs obtained from non-selected clusters, clusters containing SBE (S) or CAGA (C) motifs by MEME, MAST, and Tomtom analyses are indicated together with *p*-values and E values.

Table S3.
Occurrence of the MEME motifs in Smad2/3-binding sites obtained by ChIP-seq/chip analyses

meme	transcription factor	ID	A549-ChIP	2,535 sites	HepG2-ChIP	2,704 sites	HaCaT-ChIP	3,636 sites
			q-value	occurrence	q-value	occurrence	q-value	occurrence
HaCaT-5	FOS::JUNB	GNRTGASTCATC	0.076	100.99	0.217	26.90	0.0846	77.70
HaCaT-S4	FOSL2::JUN	RTGACTCAT	0.105	94.20	0.452	21.29	0.144	65.53
HaCaT-S75	FOS::JUND	TGADTCATC	0.135	46.55	0.368	15.87	0.157	35.50
A549-92	Gm397	GTGTGTGC	0.257	9.27	0.205	10.15	0.21	10.50
A549-C99	PRDM4	GGGGCCT	0.444	16.09	0.177	32.89	0.242	26.63
A549-C94	ASCL1	AGCTGCT	0.464	22.88	0.382	25.58	0.358	28.14
HepG2-58	Tcfe2a	ATCTGGSCT	0.498	31.99	0.495	26.51	0.496	28.00
HaCaT-C88	MTF1	TTTGCACAC	0.543	16.65	0.655	15.18	0.562	15.83
A549-27	Sox10	WCRCTGCTAKTG	0.569	14.16	1	12.90	1	13.94
A549-C62	MYC	GCACGTKSCT	0.616	15.66	0.298	21.09	0.349	18.90
HepG2-C58	ZNF306	GGCTAGSCT	0.668	16.09	0.482	18.51	0.52	18.97
HaCaT-S18	NFIX	CCGTGCCAG	0.724	22.25	0.174	79.54	0.27	55.25
A549-C51	ZNF435	GGTSTWCTG	0.743	25.05	0.674	23.76	0.614	24.85
HaCaT-21	SOX13	TCCAYTGTT	0.785	17.71	1	11.55	1	13.31
HaCaT-S46	SOX14	TAACATWKA	0.87	8.24	1	3.85	1	4.92
HepG2-S56	ONECUT1	AAATCRAT	1	17.63	1	9.60	1	12.35
HaCaT-73	Bhlha15	CCATATGW	1	8.84	1	4.62	1	6.43
HepG2-65	ZNF306	TCGAGGCYA	1	8.48	1	11.30	1	9.91
HepG2-25	E2F1	ATTGGCGCC	1	5.60	0.509	15.07	1	9.69
HepG2-37	REST	ATGGTGCT	1	2.45	1	2.04	1	2.18

Smad2/3-binding genomic regions (1,000 bp genomic sequences flanking the peak position) were obtained by ChIP-chip in HaCaT (GSE11710) and HepG2 (GSE28798) cells or by ChIP-seq in A549 (GSE51510) cells. The occurrence of transcription factor-binding motifs was analyzed by FIMO (Find Individual Sequence Occurrence, ver 5.3.0., https://meme-suite.org/meme/tools/fimo) and is shown as the number of motifs per 100 sites. R denotes G/A, S denotes G/C, D denotes G/A/T, W denotes A/T, K denotes G/T, and Y denotes C/T.

Figure S1

(A)

SMAD2/3/4-triple knockout A549 (A549-*SMAD2/3/4*TKO)

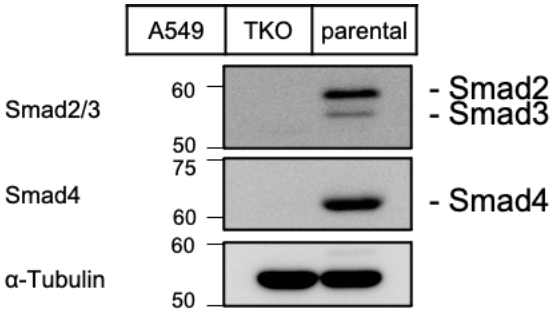

(B)

SMAD3

```
WT        TCCCTGGATGGCCGGTTGCAGGTGTCCCATCGGAAGGGGCTCCCTCATGTCATCTACTGCCGCCTGTGGCGATG
          GCCAGACCTGCACAGCCACCACGAGCTACGGGCCATGGAGCTGTGTGAGTTCGCCTTCAATATGA

allele 1  TCCCTGGATGGCCGGTTGCAGGTGTCCCATCGGAAGGGGCTCCCTCCATGGCTGTG------------------
          ----------------------------CGGGCCATGGAGCTGTGTGAGTTCGCCTTCAATATGA

           S  L  D  G  R  L  Q  V  S  H  R  K  G  L  P  P  W  L  C
        70
                                          G  P  W  S  C  V  S  S  P  S  I  *

allele 2  TCCCTGGATGGCCGGTTGCAGGTGTCCCACCGCTGGTAGCGGTGGTTTGTTTGCCGGATCAAGAG …

        70 S  L  D  G  R  L  Q  V  S  H  R  W  *
```

SMAD4

```
WT        ATTGTGCATAGTTTGATGTGCCATAGACAAGGTGGAGAGAGTGAAACATTTGCAAAAAGAGCAATTGAAAGTTTGGTAA

allele 1  ATTGTGCATAGTTTGATGTGCCAT----AAGGTGGAGAGAGTGAAACATTTGCAAAAAGAGCAATTGAAAGTTTGGTAA
            I  V  H  S  L  M  C  H     K  V  E  R  V  K  H  L  Q  K  E  Q  L  K  V  W  *
          19
allele 2  ATTGTGCATAGTTTGATGTGCCATGATGTGCCATAGACATAGACAAGG

          19 I  V  H  S  L  M  C  H  D  V  P  *
```

Figure S1. Establishment of *SMAD2/3/4*-triple knockout A549 cells

(A) Expression of Smad proteins in A549 parental and *SMAD2/3/4*-triple knockout (TKO) cells was examined by immunoblot analysis using the indicated antibodies (left). α-tubulin was used as a loading control. (B) Genomic DNA sequences of the *SMAD3* and *SMAD4* alleles in knockout cells. The sequences of the *SMAD2* alleles were reported previously [Itoh Y, Sawaguchi T, Fu H, Omata C, Saitoh

M, Miyazawa K. Indole-derived compound SIS3 targets a subset of activated Smad complexes. *J Biochem.* 2023; 173:283-291.].

Figure S2

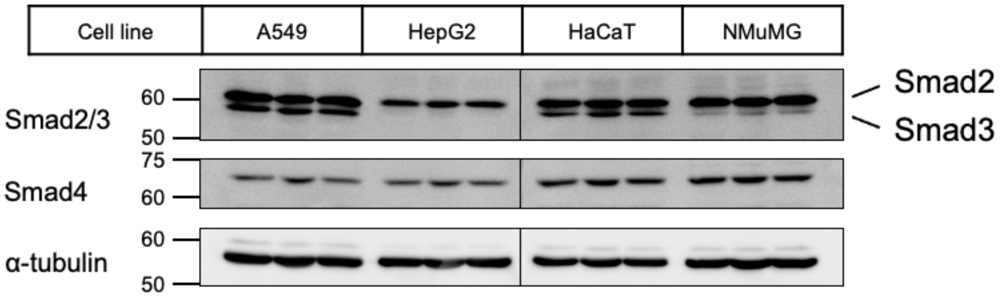

Figure S2. Expression of Smad2, Smad3 and Smad4 in A549, HepG2, HaCaT and NMuMG cells

Expression of Smad2, Smad3, and Smad4 proteins was examined by immunoblot analysis using the indicated antibodies (left). Forty μg of protein in each lysate was loaded. α-tubulin was used as a loading control.

Figure S3

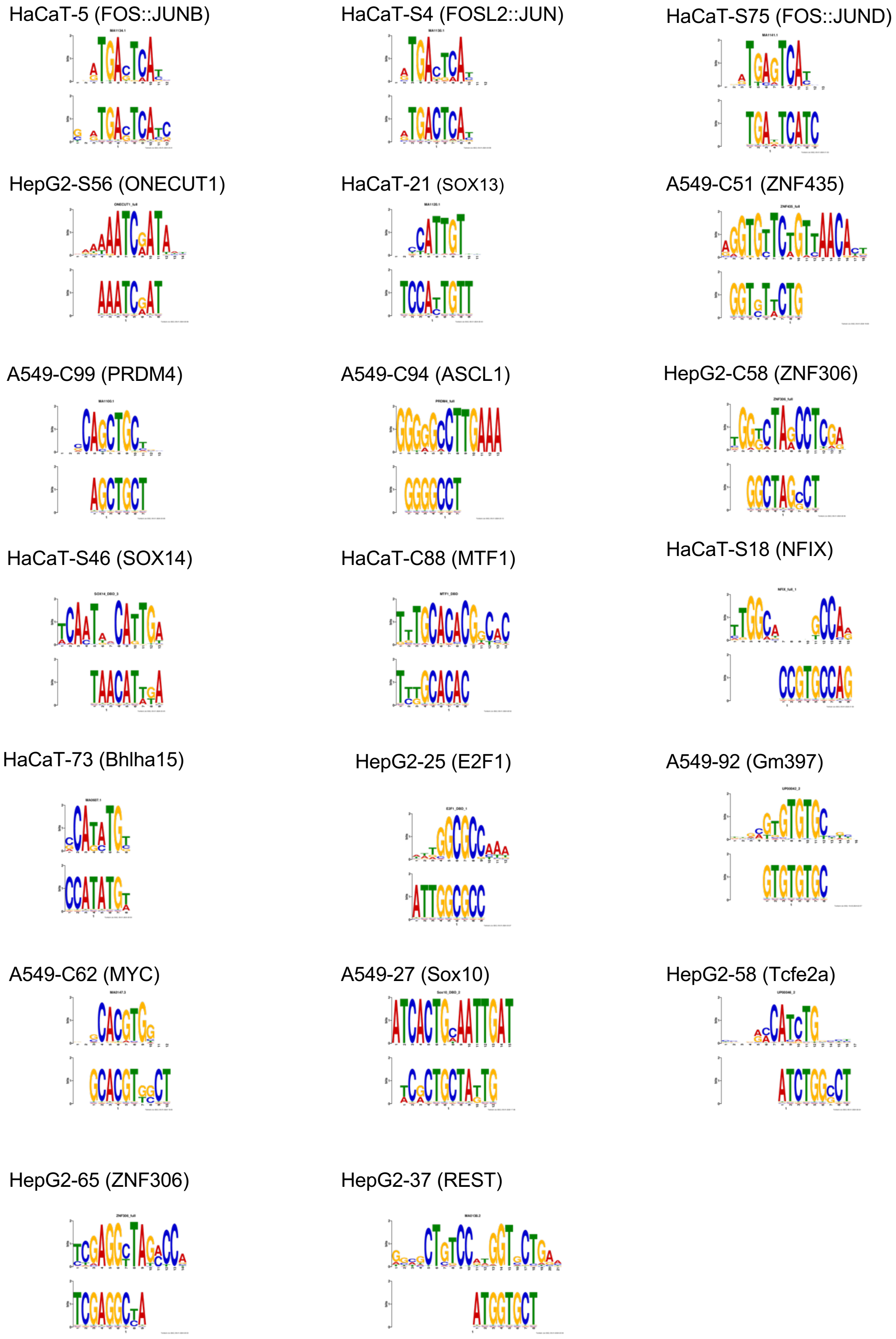
HaCaT-5 (FOS::JUNB)
HaCaT-S4 (FOSL2::JUN)
HaCaT-S75 (FOS::JUND)
HepG2-S56 (ONECUT1)
HaCaT-21 (SOX13)
A549-C51 (ZNF435)
A549-C99 (PRDM4)
A549-C94 (ASCL1)
HepG2-C58 (ZNF306)
HaCaT-S46 (SOX14)
HaCaT-C88 (MTF1)
HaCaT-S18 (NFIX)
HaCaT-73 (Bhlha15)
HepG2-25 (E2F1)
A549-92 (Gm397)
A549-C62 (MYC)
A549-27 (Sox10)
HepG2-58 (Tcfe2a)
HepG2-65 (ZNF306)
HepG2-37 (REST)

Figure S3. Transcription factor-binding motifs concentrated in Smad2/3-binding sequences obtained by CASTing analysis

Concentrated motifs in Smad2/3-binding sequences obtained by CASTing analysis are shown in the lower panels. Best match transcription factor-binding motifs from databases using Tomtom are shown in the upper panels, and the names of transcription factors are shown in parentheses.

Figure S4

(A)

number of clusters	Smad2/3 binding sequence	reporter construct
201	CAGACGATGAGTCATCAGCAGTCTAGACAGTATGAA	AP1-1
151	TTCCTAGCGATGAGTCACGCGTCTGTCTAGACACTG	AP1-2
146	CAGGGATGACTCAGCACGATGTCTAGACGGTCCGAA	
136	CAGGGCTGAGTCATGCCTCATGTCTAGACATACGAA	
125	CAGACTGATGACTCACCCCAAGTCTAGACATAGGAA	
114	TTCGTTGGCATGACTCACTCATTGTCTAGACACCTG	
108	CAGGGCGTATGACTCATCGCATGTCTAGACACAGAA	AP1-3
83	CAGCCGTGTGACTCACCTTCTGTCTAGACATATGAA	
75	CAGGGGTGACTCATCGTAATACGTCTAGACAGCGAA	
72	TTCTGATGACTCATCAATGTCTAGACGTCTCGCCTG	
68	TTCGATGAGTCATTCTTGAAGTCTAGACATAGCCTG	
63	CAGGGTGACTCACCACCGCTGTCTAGACAATACGAA	
63	TTCGGAGTAGTGTGACTCATCGGGCTGTCTAGACTG	
62	CAGGGAACATGACTCACAGCCGTCTAGACATTCGAA	
61	CAGGCCCTGATGACTCACACACTGTCTAGACGGGAA	
60	TTCGGTGACTCATCCTGTGTCTAGCCATCATGTCTG	
54	CAGTGACTCATCAATCAGTCTAGACATATCAACGAA	
45	TTCTACAATGACTCATCGCTGTCTAGACAACGGCTG	AP1-4
44	TTCGGGCTACGATGAGTCACAAGGGGTGTCTAGACTG	
43	TTCGGTGAGTCATGGCTGTCTAGACAAAGCCCTCTG	
41	TTCTCTGACTCACCTCACTGTCTAGACAGTTGGCTG	
41	TTCGTATGACTCAGGCCAATGTCTAGACGCTGGCTG	
40	TTCGGATGACTCACTGGCTGTCTAGACGCCCTGCTG	
37	TTCGTGTGACTCACAGTGGTCTAGACATGGTGTCTG	
35	CAGCGCGGCATGACTCATCGGAGTCTAGACAGCGAA	
34	TTCGGGTATTGAATGAGTCACCCCCTGTCTAGACTG	
31	TTCGTATGATGAGTCATCAGTGCCCTGTCTAGACTG	
31	TTCTGTGGGGCATGAGTCATCCTCCTGTCTAGACTG	
30	TTCGCTGCCTGATGAGTCACCTGGCCGTCTAGACTG	
29	TTCGATGACTCAAGCGGACTGTCTAGACACTG	
29	TTCGTGATGAGTCATACGCGTCTAGACAGGGCGCTG	
29	CAGCCGTGTGACTCATGGAGCGTCTAGACAGTTGAA	
28	CAGGGCATCGCGGTGAGTCATACATGTCTAGACGAA	
28	TTCGCCTGACTCATCTTTGTCTAGACATACGCCCTG	
28	TTCAAAAGGATGAGTCATTGCACAGTGTCTAGACTG	
27	TTCGCTGGTGTGCATGAGTCACCTCCGTCTAGACTG	
27	TTCAACTGTGCATGACTCATCCGGTTGTCTAGACTG	
27	TTCAGGGGGACACGTGAGTCACCACTGTCTAGACTG	
26	TTCGCAGTGATGACTCATCGCCCTGTGTCTAGACTG	
26	TTCGTGCGTATCTGAGTCATACGACTGTCTAGACTG	
26	TTCAATGAGTCATGGTGTTGTCTAGACGGCAGTCTG	
26	TTCGATGTATGAGTCACCCCTCTTGTCTAGACGCTG	
25	TTCGTGGAATGACTCATCGCTCTTATGTCTAGACTG	
24	CAGGGCGCTGAGTCATGGTCGTCTAGACACATCGAA	
24	TTCGCGTGTGTGGGTGAGTCATCTCTGTCTAGACTG	
23	CAGCGGACCGTCTGACTCAGACAGTGTCTAGACGAA	
22	TTCGTCTGACTCACCAGTATGTCTAGACGTGGGCTG	

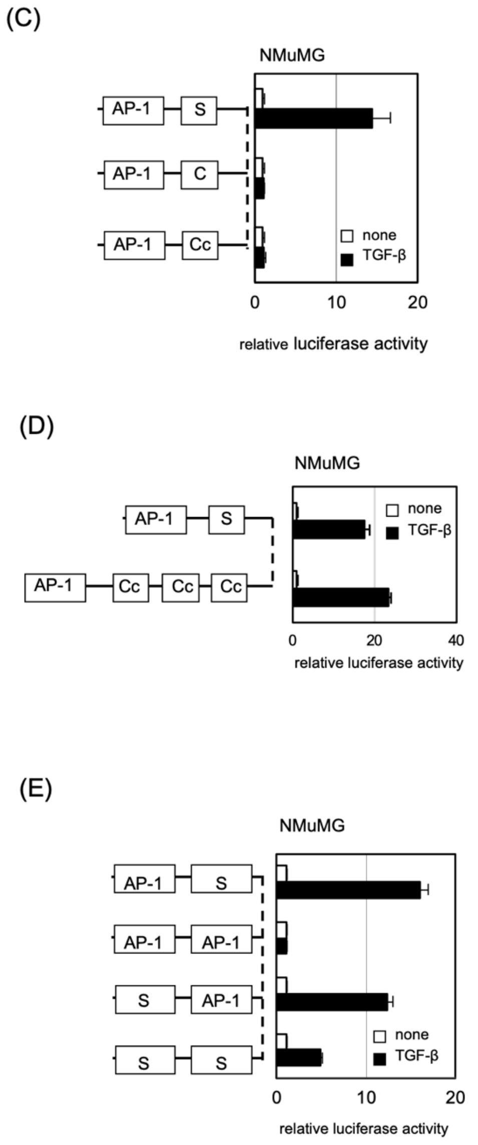

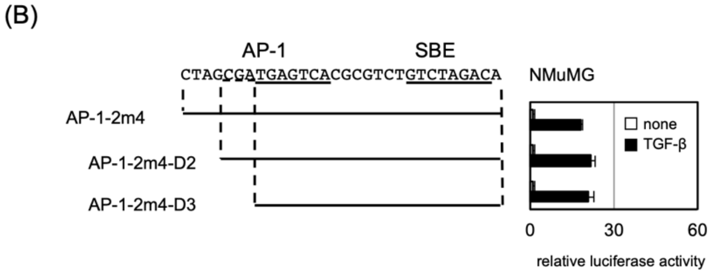

Figure S4. Transcriptional activation of the AP-1 binding motif and SBE composite reporters in response to TGF-β stimulation

(A) SBE/AP-1 motif composite sequences obtained by the CASTing analysis in HaCaT cells are listed in descending order of cluster sizes (shown in the left). Total reads: 261,925. The names of the reporters used in Figure 3 are shown in the right. 5′-TTC/GAA-3′ and 5′-CTG/CAG-3′ are common flanking sequences of the library. SBE and the AP-1 binding motifs are colored *red* and *purple*, respectively. (B-E) Reporter activities were measured in NMuMG cells. (B) Effects of deletions in a flanking region upstream of the AP-1 motif. (C) Substitution of the SBE (S) with a single CAGA (C) or its complementary CAGA (Cc) motif attenuated the reporter activity. (D) Substitution of the SBE motif with a triple-CAGA motif maintained the reporter activity. (E) Substitution of the SBE or the AP-1 motif with the other attenuated the reporter activity. Data are shown as fold induction by TGF-β stimulation (1 ng/mL for 18 h). Error bars represent S.D. from three experimental replicates.

Figure S5

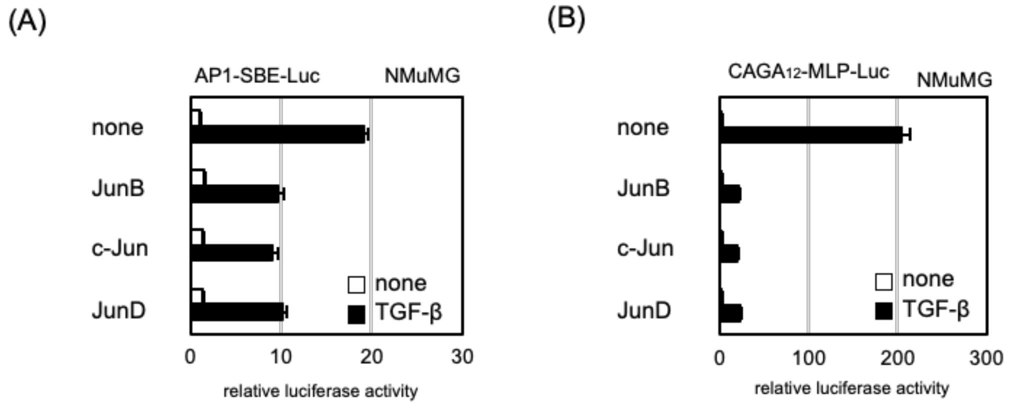

Figure S5. Effects of overexpression of Jun family proteins on AP1-SBE-Luc or $CAGA_{12}$-MLP-Luc activities in response to TGF-β stimulation

(A) AP1-SBE-Luc. (B) $CAGA_{12}$-MLP-Luc. Reporter activities were measured in NMuMG cells. Jun family proteins were transiently overexpressed. Data are shown as fold induction by TGF-β stimulation (1 ng/mL for 18 h). Error bars represent S.D. from three experimental replicates.

Figure S6

```
1. HepG2-S56(ONECUT1)        AAATCRAT
                       TCTAA AAATCGAT ATGTCTAGACTTGGCCCGGCT
                          TC AAATCGAT ATGTCTAGACACACCGTCTGGGCT *
   AGCCCAGACACCGTCTAGACAAG AAATCAAT GGA

2. HaCaT-21(SOX13)       TCCAYTGTT
       TCGTCTAGACACTATC TCCATTGTT GTCTAGACT
                        TCCATTGTT TTCCTGCGAGCCCCAGTACTAGACT
                        TCCACTGTT AATGTCTAGACAGACTGTCTGGCCT *

3. A549-C51(ZNF435)        GGTSTWCTG
                       TCC GGTGTTCTG CTGTCTAGACACTGTCTGGCCT *
  AGTCTAGACATGGTCCAGACAAG GGTGTACTG GA
  TCCGTCTAGACAGTGTCTGGCTG GGTCTTCTG CT

4. A549-C99(PRDM4)                                  GGGGCCT
                     TCGTGTCTAGACAGGCACATGTCTGGC GGGGCCT
                     TCGTCTAGACGTCTGTCTGGTTTATTG GGGGCCT
                     TCCTGTCTAGACACTGTCTGGATCCTT GGGGCCT *

5. A549-C94(ASCL1)                   AGCTGCT
                 TCCTAGTGTCTAGACAG AGCTGCT GTGTCTGGCT
                            AGACGC AGCTGCT GCCAGACAGTGTCTAGACCGA *
      TCGGTCCTGTCTAGACGGTGTCTGGTA AGCTGCT

6. HepG2-C58(ZNF306)                GGCTAGSCT
        TTCAATGTCTAGACGTTCTGTCTGGT GGCTAGCCT G
        TTCGTCTAGACACTTGTCTGGCGTTC GGCTAGCCT G *
                            CAGGGC GGCTAGGCT TCCAGACACAGTCTAGACGAA

7. HaCaT-S46(SOX14)               TAACATWKA
                               TC TAACATTGA TGTCTAGACACTGTCTGGCCGCT *
                        TCGCGGGCG TAACATTTA TGTCTAGACAGTGTCT
      AGCCGTGCCAGACAGTGTCTAGACA TAACATGA

8. HaCaT-C88(MTF1)                 TTTGCACAC
                     TCTGGTGTCTGGT TTTGCACAC GTCTAGACAGCT *
                   TCATTTGTGTCTGGT TTGGCACAC TGTCTAGACT
                       AGTCTAGACAG TCTGCACAC TCCAGACAGCATGA

9. HaCaT-S18(NFIX)                 CCGTGCCAG
                        TCGATAAGGT CCGTGCCAG ACATTGTCTAGACT
                                AG CCGTGCCAG ACAGTGTCTAGACATAACATAGA
                             AGCGT CCGTGCCAG ACACCGTCTAGACACAACGA
                           TCGAAAG CCGTGCCAG CCTGTCTAGACACTGTCT *

10. HaCaT-73(Bhlha15)              CCATATGW
          AGTCTAGACAGTGTCTGAAGGC CCATATGT GCGA
                                TC CCATATGT TCCAGACGTGTGTCTAGACACCCT
                                AG CCATATGA ACTAGACAGTGTCTAGACAGACGA *
```

Figure S6 (continued)

```
11. HepG2-25(E2F1)                       ATTGGCGCC
          TCGTAGACGTTCGTCTAGACAC ATTGGCGCC CCT *
                          TCGTTC ATTGGCGCC TCAATATGGCCTCTAGACT
        TCGAGTGTCTAGACGTCTGGCGGG ATTGGCGCC T

12. A549-92(Gm397)                       GTGTGTGC
       TCGTCTAGACAGTGTCTGGTCCCGC GTGTGTGCT
       TCGCAGACATTGTCTAGACAGGTGT GTGTGCGCT
       TCGAGTGTCTAGACAATGTCTGGGC GTGTGTGCT *

13. A549-C62(MYC)                        GCACGTKSCT
        TCGCGTCTAGACGGTGTCTGGACG GCACGTGGCT *
        TCTGTGTCTAGACATGTCTGGCTG GCACGTTGCT
        TCTGTCTAGACAATGTCTGGTCCT GCACGTGCCT

14. A549-27(Sox10)                     WCRCTGCTAKTG
                                       TCGCTGCTATTG TCTAGACATGTCTGGTCTGGCT *
                      AGTCTAGATTCGCGC TCGCTGCTAGTG TCGATGA
                                  TCA ACACTGCTATTG TGTGACGCGGCAAGGAGCT

15. HepG2-58(Tcfe2a)                      ATCTGGSCT
       TCTCTAGACATAGTCTAGACTGGC ATCTGGCCT *
      TCGTCTAGACATAGTCTAGACTGGC ATCTGGCCT
                        TCGCTCT ATCTGGGCT GGTCGTGGTTCCACATCT

16. HepG2-65(ZNF306)                 TCGAGGCYA
                                     TCGAGGCTA AAAGTCTAGACGGTCGTCTGGAGCT *
                                     TCGAGGCTA TATGTCTAGACAGTGGCTGGACGCT
                                     TCGAGGCCA CCTTCTGGCCAGGCTGCTCTAGACT

17. HepG2-37(REST)                     ATGGTGCT
    TCCATGTCTAGACAGTGTCTAGACGA ATGGTGCT
    TCGCTAGACACTGTCTAGACATGTGG ATGGTGCT *
    TCTATGTCTAGACAGTGTCTAGACGA ATGGTGCT
```

Figure S6. Concentrated sequences containing 17 selected motifs presented by MEME

Concentrated sequences containing 17 selected motifs presented by MEME (shown in bold) are listed. 5′-TC/GA-3′ and 5′-CT/AG-3′ are common flanking sequences of the library. The top predicted transcription factors from each motif matrix identified using Tomtom are shown in parentheses. SBE (GTCTAGAC) and CAGA motifs (TGTCTGG) are colored *red* and *blue*, respectively. Sequences used for reporter construction are marked with asterisks. R denotes G/A, S denotes G/C, D denotes G/A/T, W denotes A/T, K denotes G/T, and Y denotes C/T.
